# Hydroxide Diffusion in Functionalized Cylindrical Nanopores as Idealized Models of Anion Exchange Membrane Environments: An Ab Initio Molecular Dynamics Study

**DOI:** 10.1021/acs.jpcc.2c05747

**Published:** 2023-02-02

**Authors:** Zhuoran Long, Mark E. Tuckerman

**Affiliations:** † Department of Chemistry, New York University, New York, New York10003, United States; ‡ Courant Institute of Mathematical Science, New York University, New York, New York10012, United States; ¶ NYU-ECNU Center for Computational Chemistry at NYU Shanghai, 3663 Zhongshan Road North, Shanghai200062, China

## Abstract

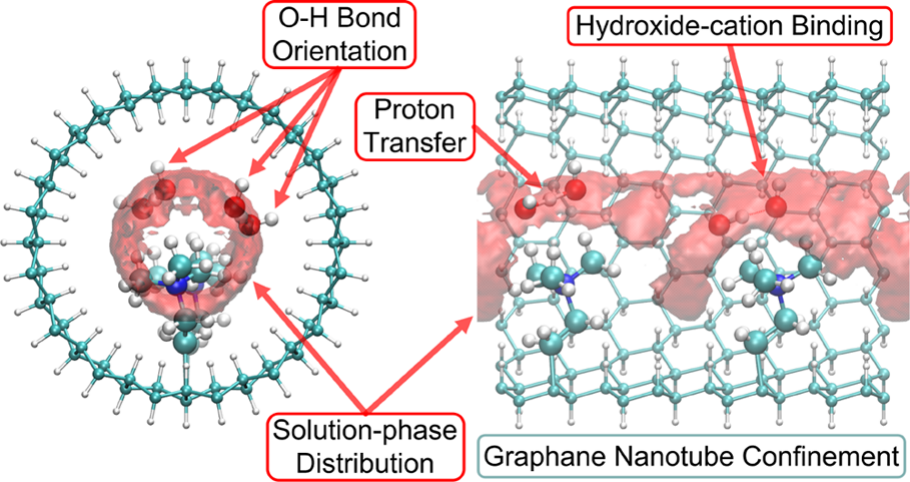

Anion exchange membranes (AEMs) have attracted significant interest for their applications in fuel cells and other electrochemical devices in recent years. Understanding water distributions and hydroxide transport mechanisms within AEMs is critical to improving their performance as concerns hydroxide conductivity. Recently, nanoconfined environments have been used to mimic AEM environments. Following this approach, we construct nanoconfined cylindrical pore structures using graphane nanotubes (GNs) functionalized with trimethylammonium cations as models of local AEM morphology. These structures were then used to investigate hydroxide transport using ab initio molecular dynamics (AIMD). The simulations showed that hydroxide transport is suppressed in these confined environments relative to the bulk solution although the mechanism is dominated by structural diffusion. One factor causing the suppressed hydroxide transport is the reduced proton transfer (PT) rates due to changes in hydroxide and water solvation patterns under confinement compared to bulk solution as well as strong interactions between hydroxide ions and the tethered cation groups.

## Introduction

Anion exchange membrane (AEM) fuel cells (AEMFCs) rank among the cleanest low-cost electrochemical device technologies. The low cost is a result of the fact that AEMFCs operate in an alkaline environment, which obviates the need for precious metal catalysts^1−9^ and gives AEMFCs an advantage over present proton exchange membrane fuel cells. Early versions of AEMFCs were used by NASA to power onboard systems during the Apollo and space-shuttle missions.^10^ However, the need to enhance both hydroxide conductivity and chemical stability in AEMFCs remains an impediment to their widespread adoption. Designing AEMs with high hydroxide conductivity and chemical stability requires a detailed understanding of the hydroxide transport mechanism in geometries reflective of AEM environments.

Molecular dynamics (MD) is a powerful tool for studying the atomistic mechanisms that govern hydroxide transport mechanisms in both bulk and nanoconfined environments. Hydroxide diffuses structurally (also known as the Grotthuss mechanism) via individual sequential proton transfer (PT) events initiated by presolvation of the hydroxide ion.^11−17^ Previous MD studies of hydroxide transport in AEM systems employed reactive force fields to achieve chemical bond-breaking and forming in PT.^5,18−22^ A multistate empirical valence bond (MS-EVB) study of a poly(vinyl benzyltrimethylammonium) (PVBTMA) system indicated that the hydroxide transported within the first solvation shell of trimethylammonium (TMA) cations with a greater contribution from vehicular mechanism. The regularly spaced TMA cations observed in this system form overlapping first solvation shell regions, allowing hydroxide to transport from the solvation shell of one cation to the other. In comparison, a series of ReaxFF simulations carried out in functionalized poly(phenylene oxide) (PPO) systems suggested that the structural diffusion mechanism is dominant and, in particular, is critical for hydroxide ions to pass through bottlenecks of water channels where vehicular diffusion is associated with high energy barriers needed to shed solvating waters upon entering these bottleneck regions.^18−21,23−26^ The dominance of structural diffusion is also supported by various experimental measurements^27^ as well as overly low vehicular diffusion coefficients of hydroxide from nonreactive force field MD simulations.^4,18−21,28−32^

Ab initio MD^33−35^ (AIMD) simulations have proved enormously successful in predicting hydroxide diffusion constants, mechanisms, and kinetics in aqueous solution and in revealing both the presolvation concept and the dynamical hyper-coordination picture of hydroxide transport.^13,14,17,36−40^ In an AIMD simulation, nuclear motion is generated from Newton’s second law of motion using forces generated “on the fly” from electronic structure calculations. The high computational overhead and O(*N*^3^) system size scaling of AIMD precludes modeling AEMs at the mesoscale. On the other hand, nanoconfined environments have been utilized in both experimental and theoretical studies to model polymer architectures and nanoporous materials for use in electrochemical devices.^23−26,41−51^ Materials with cylindrical pores, in particular, have attracted interest for their ability to enhance diffusion in proton-conducting liquids.^41,44,45,50,52−54^ In addition, polymers such as PPO can support morphologies containing tight pores for different linker lengths to the TMA cations.^55−57^ In order to capture the effect of the AEM nanoconfined environment on hydroxide diffusion mechanisms, idealized geometries such as graphane bilayers and carbon nanotubes to which cationic functional groups are attached have proved successful.^23−26,49,51^ Our previous work employed graphane bilayer structures as an idealized mimic of a lamellar AEM morphology.^23−26,49,51^ In this work, we consider an idealized mimic of the type of nanopore that might arise in bicontinuous morphologies by employing graphane nanotube (GN) structures functionalized by cations attached to the inside of the tube via short linkers. AIMD simulations are performed in order to probe the influence of the cylindrical confinement and cation placement. The simulations indicate that although dominated by structural diffusion, the hydroxide transport is suppressed in these confined systems compared to bulk solution. We attribute the suppressed hydroxide transport to the decreased PT rate due to the high barriers associated with the presolvation under nanoconfinement, as well as strong hydroxide–cation interactions.

## Methods

**Figure 1:**
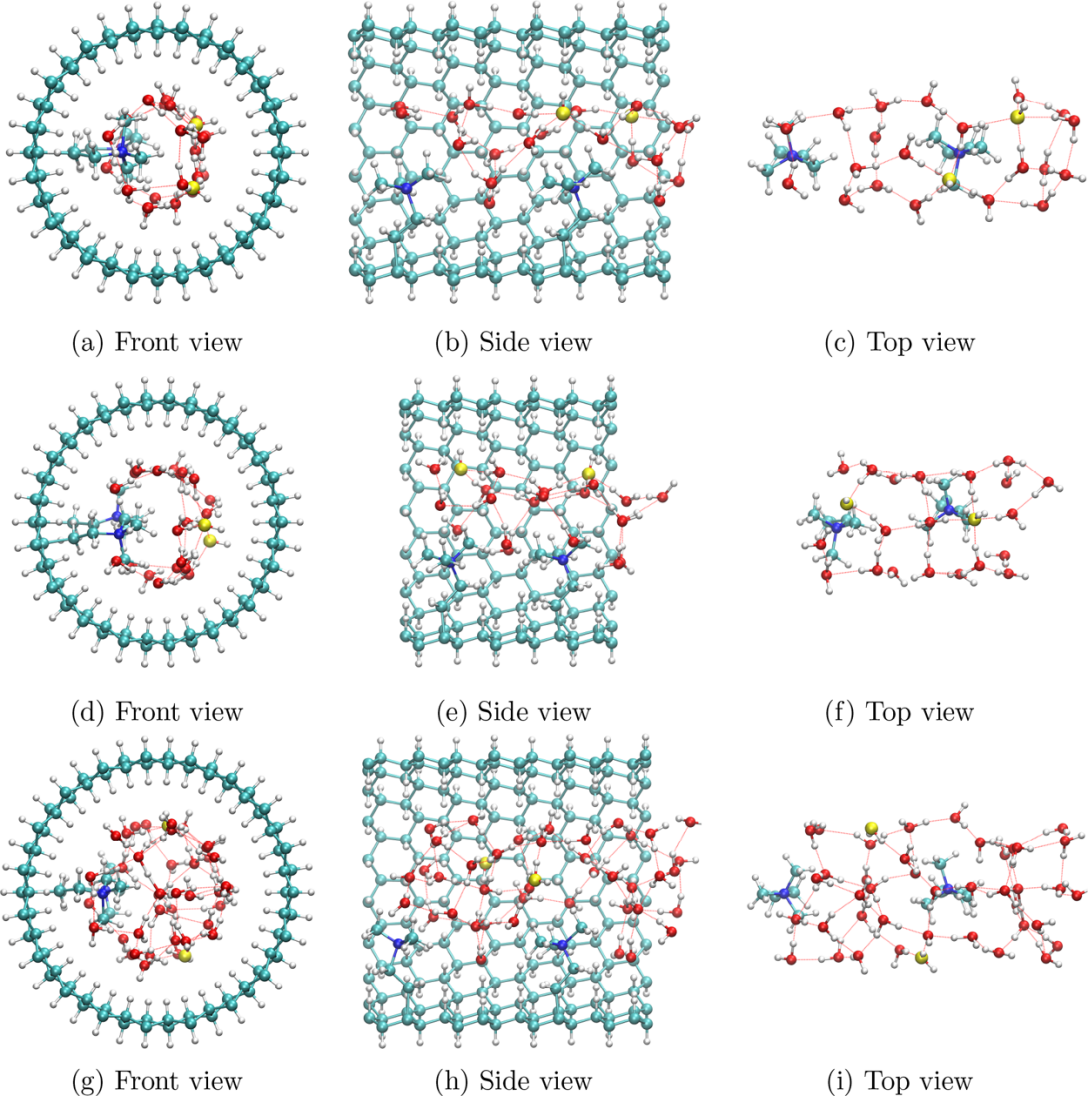
Snapshots (a–c) of GN(16,0), (d–f) of GN(17,0), and (g–i) of GN(19,0) system. The snapshots are shown from (a,d,g) front view (*z*), (b,e,h) side view (ϕ = −90°), and (c,f,i) top view (ϕ = 0). The front part of the GN in the side view and the whole GN in the top view are hidden for visual clarity. Only one periodic replica is present in each snapshot. Color maps are: hydrogen: white, oxygen: red, carbon: cyan, nitrogen: blue, and hydroxide oxygen atoms: yellow. Hydrogen bonds are shown as red dashed lines.

Snapshots depicting our GN models are provided in Figure 1, and system parameters are listed in Table 1. Within this model, zigzag GNs GN(*n*,0) mimic the hydrocarbon backbone in a local cylindrical pore structure in an AEM. The GNs are aligned along the*z* axis with periodic boundary conditions applied in this direction to form channels of infinite length for the nanoconfined alkaline aqueous solutions. Two evenly spaced TMA cations are tethered to the GN wall with −CH_2_–CH_2_– linkers, and two hydroxide ions are added to the solution phase to achieve electrical neutrality. The cation separation *d*_cat_ corresponds to the distance between cation tethering points on the GN wall, which also determines the GN length *z*_GN_. The water content of GN(16,0) is set to λ = 10, where λ is the ratio of the number of water molecules to the number of cations. The GN(17,0) has the same water content but a shorter cation separation. Its GN radius (determined by the value of *n*) is increased to compensate for the reduced GN length. Nevertheless, the GN(17,0) ultimately has a higher solution density than the other two systems. Finally, GN(19,0) has a higher water content (λ = 20) and consequently a larger GN radius, while its GN length is kept identical to the GN(16,0). The GN radii correspond to microphase-separated water channels observed in AEM morphology studies,^2,21,22,32,58−62^ which have size distributions in the range 4–10 Å in TMA-functionalized PPO.^19−21^ The cation separations correspond to the cation–cation radial distribution functions (RDFs) which have their first peaks at 6–7 Å and second peaks at about 9 Å.^2,5,18,22,59−61^ The densities of the solution phase ρ_sol_ are calculated from the available volumes inside the GN confinements *V*_conf_ using the PLATON package^63^ with the probe radius kept at the default value of 1.2 Å. This approach was previously employed to calculate channel volumes in other systems such as metal-organic frameworks^64^ and molecular sieves.^65^

**Table 1:** System Parameters of Confined Graphane Nanotube (GN) Systems, Including Water Content (λ), Nanotube Radius, Nanotube Periodic Length (*z*_GN_), Cation Separation (*d*_cat_), Available Volume Inside the GN Confinement (*V*_conf_), Solution-Phase Density (ρ_sol_), and Average Temperature (*T*) of NVE Trajectories

system name	λ	radius (Å)	*z*_GN_	*d*_cat_ (Å)	*V*_conf_ (Å^3^)	ρ_sol_ (g/cm^3^)	*T* (K)
GN(16,0)	10	6.198	17.43136	8.72	683	1.06	291.2
GN(17,0)	10	6.599	13.07352	6.65	592	1.22	304.2
GN(19,0)	20	7.400	17.43136	8.72	1323	1.05	313.4

AIMD simulations of these confined systems were performed with the CPMD code.^66^ The electron structure is described at the level of density functional theory (DFT)^67−69^ using the BLYP generalized gradient functional.^70,71^ Despite the approximations inherent in the use of the BLYP functional for aqueous systems, our choice of this functional for this study is based on several considerations. First, previous studies with it demonstrated its ability to produce correct hydroxide transport mechanisms in bulk aqueous solutions^14,36−38,72−74^ and to yield new and testable predictions of hydroxide transport under nanoconfinement that have been confirmed experimentally.^26,75^ Moreover, when used with a converged basis set, its description of the structural and dynamical properties of bulk water are reasonable.^76−78^ In order to connect the present study with our previous work, we, therefore, opted to employ the BLYP functional again here; however, in recognition of the need for a large basis set, we used a cutoff value of 80 Ry for the plane wave basis set. We are, however, aware of the caveats associated with the use of this functional and will address improvements for future studies in the Conclusions section. In this study, dispersion interactions were included via the dispersion-corrected atomic core pseudopotentials (DCACP) approach.^79,80^ The Car–Parrinello algorithm^81^ is used with a fictitious electron mass of 600 a.u. and a time step of 4 a.u. All GN carbon and hydrogen coordinates were kept fixed throughout the simulations. The deuterium mass was used for all hydrogen atoms to reduce the importance of nuclear quantum effects. After wave function minimization, each system was heated to approximately 300 K and then equilibrated in the NVT ensemble with massive Nosé–Hoover chain thermostats^82^ (12 ps for GN(16,0), 13 ps for GN(17,0), and 10 ps for GN(19,0)). Following equilibration, production runs were performed in the NVE ensemble (87 ps for GN(16,0), 109 ps for GN(17,0), and 73 ps for GN(19,0)). These simulation settings are consistent with our previous studies in systems confined between graphane bilayers.^23−26,49,51^

## Results and Discussion

### Diffusion Coefficients of Hydroxide

The diffusion coefficients of hydroxide ions serve as the microscopic indicator of hydroxide conductivity. Structural diffusion causes the identity of the hydroxide to change with each PT reaction. Thus, in order to identify hydroxide ions in each AIMD step, each solution-phase hydrogen atom is assigned to its closest oxygen atom. Oxygen atoms with only one assigned hydrogen are recognized as hydroxide, for which the oxygen atom is denoted O* and the corresponding hydrogen as H*. Oxygen atoms with two assigned hydrogen atoms are water oxygen atoms O_W_ with hydrogen atoms denoted H_W_.

Hydroxide diffusion coefficients are calculated from the mean square displacement (MSD) of the O* coordinates, including all changes of O* identity due to PT (see Figure S1 of the Supporting Information). The results, together with water O_W_ diffusion coefficients (corrected for finite-size effects,^77,86^) are given in Table 2. The GN(19,0) system has the highest hydroxide and water diffusion coefficients, while the GN(17,0) system has the lowest hydroxide and water diffusion coefficients. Note that even the highest hydroxide and water diffusion coefficients in GN(19,0) are still smaller than the previously reported values in bulk aqueous solution obtained from experiments^14,38,83^ and AIMD simulations with the same functional.^74,77^ The GN(16,0) system with a lower water content (λ = 10) has a smaller water diffusion coefficient than the GN(19,0) system, but the hydroxide diffusion coefficients are close in these two systems. This retention of hydroxide diffusivity under decreased water diffusivity suggests a hydroxide transport mechanism dominated by structural diffusion in GN(16,0). The further reduced hydroxide diffusivity and water diffusivity in GN(17,0) results from the high solution-phase density in this system.

**Table 2:** Diffusion Coefficients of Hydroxide and Water

system name	*D*(O*) (Å^2^/ps)	*D*(O_W_) (Å^2^/ps)	*D*(O*)/*D*(O_W_)
GN(16,0)	0.10	0.037	2.7
GN(17,0)	0.044	0.019	2.3
GN(19,0)	0.11	0.080	1.4
bulk, AIMD^74,77^	0.45	0.132	3.4
bulk, experiment^14,38,83^	0.312	0.186^a^	1.7

a Measured from fully deuterated pure water without hydroxide.^14,38,84,85^

### Structure of the Solution Phase under Nanoconfinement

**Figure 2:**
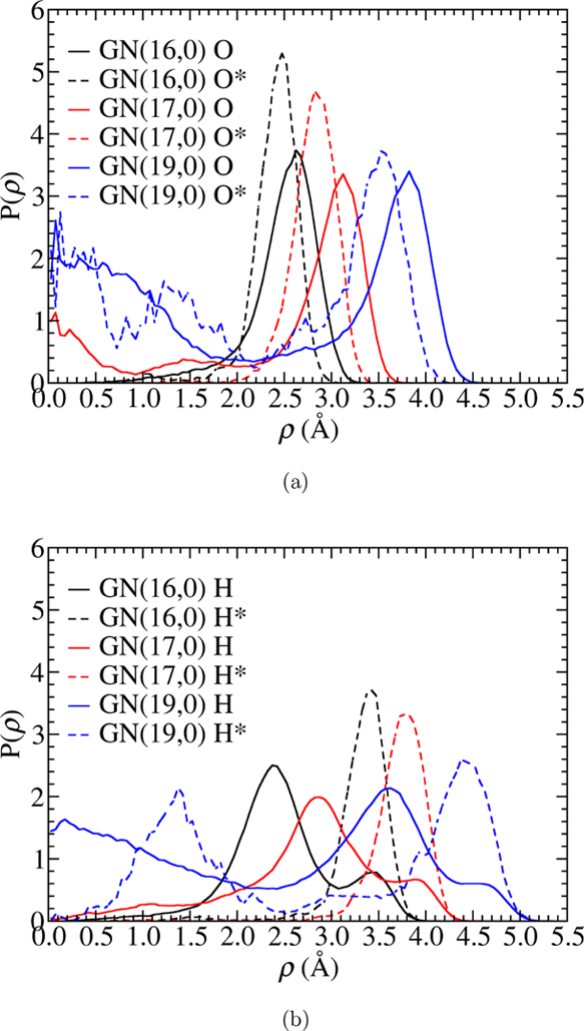
Atom distributions of (a) all oxygen atoms (O) and the hydroxide oxygen atoms (O*) and (b) solution-phase hydrogen atoms (H) and hydroxide hydrogen atoms (H*) along the GN radius ρ. The distributions are calculated as the relative numerical density of corresponding species.

Understanding the solution-phase structure under nanoconfinement is fundamental for further analysis of the hydroxide transport mechanism. The geometrical analysis will make use of standard cylindrical coordinates (ρ, ϕ, *z*), where ρ is the radial coordinate, ϕ is the azimuthal angle, and *z* is the coordinate along the length of the GN. Distributions of oxygen and hydrogen atoms in Figure 2 are calculated using 1 (below), which prescribes the probability density as a function of the cylindrical radius ρ (ρ = 0 corresponds to the GN axis):
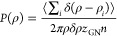
1The bracket represents the ensemble average over all configurations. The summation is taken over all the corresponding atoms. The bin width for the statistics is δρ = 0.05 Å. *z*_GN_ is the periodic length of the GNs. *n* is the numerical density of the corresponding species in the solution phase and calculated with the available volume inside the GN (*V*_conf_). Complementary analysis of oxygen distributions along the ϕ and *z* coordinates is provided in Section S2 of the Supporting Information.

In Figure 2a, the oxygen distribution peaks at ρ > 2 Å in all three systems suggest that the solution forms cylindrical layers near the GN wall. The radii of these cylindrical layers reflected in the peak positions increase with GN radius. Similar cylindrical layer structures were also observed for pure water^87^ and aqueous acidic solutions^88,89^ confined in carbon nanotubes, and they also closely resemble the layered structures seen in our confined systems between graphane bilayers.^24,26,49^ However, since we explicitly included cations in our nanoconfined system, the solution phase is mostly located in the region of – 90° < ϕ < 90°. In other words, the cylindrical layers are incomplete. Oxygen distributions are also observed at small ρ values (near the GN axes) in GN(17,0) and GN(19,0). As illustrated in Figure 1d,1g, these oxygen densities do not correspond to cylindrical structures. The main peaks of the hydrogen density distribution reside slightly inside the corresponding oxygen cylindrical layers. The small peaks or shoulders in the hydrogen distribution outside the oxygen cylindrical shell (ρ =3.5 Å for GN(16,0), 3.9 Å for GN(17,0), and 4.7 Å for GN(19,0)) suggest dangling hydrogen atoms that form no hydrogen bonds (HBs) but instead point toward the GN wall.

**Figure 3:**
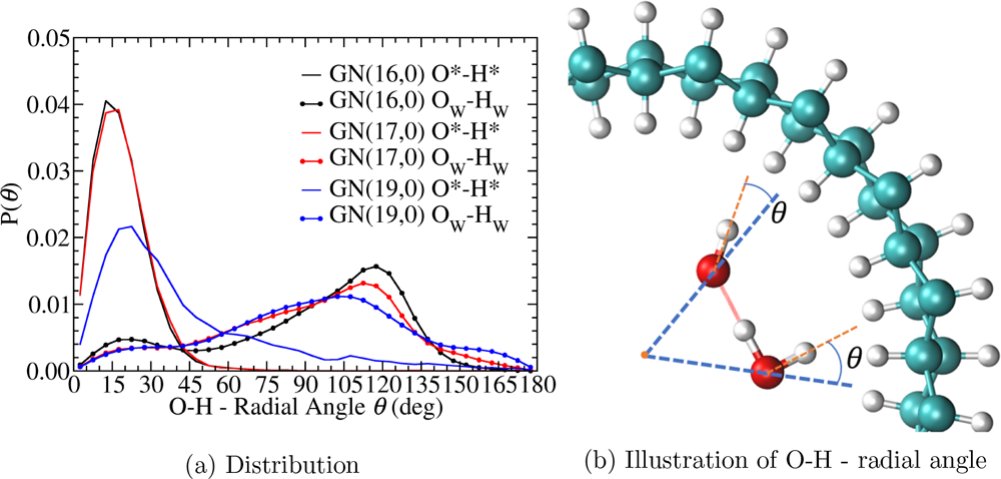
(a) Probability density distributions of the angle θ between O–H bonds and the radial vector past through the oxygen as illustrated in (b). O*–H* refers to the O–H bond of hydroxide, and O_W_H_W_ refers to the two O–H bonds of water.

While there is clear overlap between the O* and O distributions, those of O* all shift toward the center of the GN (lower ρ). The hydroxide hydrogen H* distribution peaks are in the same range as the dangling hydrogen atoms. Moreover, it is only in the GN(19,0) system that O* and H* have distributions inside the cylindrical layers. The shifted O* distribution peaks can be explained by the O*–H* orientation, which lies preferentially along the radial direction. The orientation of an O–H bond relative to the radial direction is described by the angle θ between the O–H bond and the radial vector passing the oxygen (Figure 3b). The probability density distributions of this angle have peaks at small values (0–45°) for hydroxide O*–H* in GN(16,0) and GN(17,0), indicating that the O*–H* bond of hydroxide aligns preferentially along the radial direction and points toward the GN wall. In comparison, distributions of this angle for water O_W_–H_W_ appear as flat shoulders (or a small peak for GN(16,0)) at the same range. This orientation of the dangling hydroxide O*–H* bond pointing toward the interface was also observed in hydroxide solutions confined between mackinawite sheets^90^ and at hydrophobic interfaces.^91−94^ This phenomenon has been attributed to the hydrophobic nature of the hydroxide H*.^91,94^ In the next section, we will show that this radially aligned orientation and the absence of donating HBs (dangling hydrogen) of this O*–H* bond ultimately affect the hydroxide PT mechanism.

### Detailed Mechanism of Hydroxide PT Reaction under Confinement

**Figure 4:**
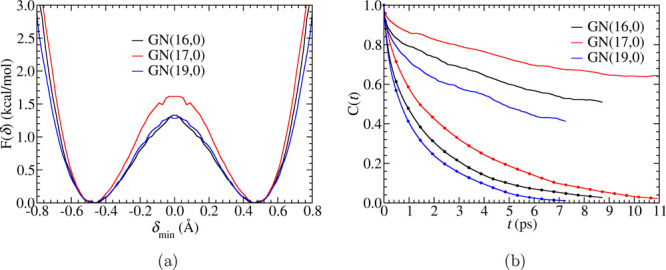
(a) Free energy profiles of proton transfer and (b) protonation population correlation functions. In (b), the continuous population correlation functions *C*_*c*_(*t*) are plotted as solid lines with dots, and the intermittent population correlation functions *C*_*i*_(*t*) are plotted as simple solid lines.

PT is the fundamental step in the structural diffusion process of hydroxide ions, and therefore, the PT mechanism affects the hydroxide transport rate. In Figure 4a, the free energy of PT reaction is calculated as *F*(δ_min_) = −*k*_*B*_*T*ln *P*(δ_min_). The reaction coordinate is defined as δ = |*r*(O_*a*_H) – *r*(O_*b*_H)|, for PT along the O_*a*_–H–O_*b*_ HB. δ_min_ is the minimum over all δ values among the HBs accepted by a hydroxide anion. The HB with δ_min_ is designated as the most active HB in the system, meaning that PT through this HB is most likely. The water oxygen in this HB is denoted by O_W_^(1*)^. A small δ_min_ (< 0.1 Å) corresponds to a proton that is nearly equally shared between the two oxygen atoms, i.e., the PT transition state. A large δ_min_ (> 0.5 Å) value indicates that the proton is attached to one of the oxygen atoms and can be considered as a PT resting state. The free energy profiles are symmetrized about δ_min_ = 0, and minima are shifted to zero. All resulting free energy profiles are double-well shaped with minima at δ_min_ = ± 0.46 Å and barriers at δ_min_ = 0. GN(19,0) and GN(16,0) have similar barrier heights of 1.3 kcal/mol, while the barrier of GN(17,0) is higher (1.6 kcal/mol). These free energy barriers are more than twice the thermal energy *k*_B_*T* = 0.596 kcal/mol at 300 K.

The reaction rate of PT can be gleaned from the protonation population correlation functions.^39,40^ Two population functions, *h*(*t*) and *H*(*t*), are defined as follows: *h*(*t*) = 1 if the investigated oxygen is the hydroxide O* at time *t* and otherwise 0, and *H*(*t*) = 1 if the investigated oxygen is hydroxide O* at time 0 and keeps its identity during the time period from 0 to *t* and otherwise 0. With these population functions, we can define an intermittent (history independent) population correlation function as
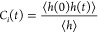
2and a continuous (history dependent) population correlation function as
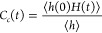
3The function *C*_*i*_(*t*) gives the probability that an oxygen that was O* at *t* = 0 becomes O* again at time *t* independent of any changes in identity between 0 and *t*. The function *C*_*c*_(*t*) gives the probability that O* retains its identity for a time *t*. One process contributing significantly to *C*_*c*_(*t*) is proton rattling, i.e., the proton shuttles back and forth along the same HB, which contributes nothing to overall hydroxide transport. The long timescale behavior of the hydroxide identity decay (slow processes) are contained in the intermittent population correlation function *C*_*i*_(*t*). As shown in Figure 4b, for both the continuous and the intermittent population correlation functions, GN(17,0) has the slowest hydroxide identity decay, while GN(19,0) has the fastest. This ordering of the hydroxide identity decay rate (PT rate) is consistent with the ordering of the hydroxide and water diffusion coefficients in Table 2.

The PT rate is closely related to the underlying molecular mechanism. In the dilute bulk aqueous solution, PT of hydroxide follows the dynamic hyper-coordination mechanism.^13,14,17,38^ Hydroxide at the PT resting state is preferentially hyper-coordinated, i.e., accepting four HBs from solvating water molecules and occasionally donating one. During a PT reaction, the hydroxide loses an accepting HB and strengthens the donating HB. Thus the hydroxide forms a water-like tetrahedral solvation structure with three accepting HBs and one donating HB and is, thus, properly “pre-solvated” for a subsequent PT reaction. However, PT via the “PT-active complex” (a tetrahedrally coordinated hydroxide and proton-donating water) at the transition state is not the only feasible pathway, and, in fact, changes in the local environment dictate which pathway dominates the PT process. Recent work reported simulations of aqueous NaOH solutions using a neural network potential.^95^ The “most important”, or most active, presolvation structure, defined as the structure with the highest PT rate and the lowest free energy barrier, changes with NaOH concentration. These authors attributed this observation to changes in the solvation configurational distributions of hydroxide and water with increasing NaOH concentrations. Accordingly, the presolvation process, which requires a change from the most probable solvation configuration to the most active configuration, was achieved by changes in accepting HBs, donating HBs, or coordinating Na^+^ cations of the hydroxide or the proton-donating first solvation shell water. Furthermore, we noticed that in all the most active presolvated PT complexes, hydroxide and proton-donating water were similarly solvated and produced symmetric PT free energy profiles.

Here we will show that similar to the NaOH concentration dependence, the confined environment of the GN also modulates the solvation configurational distributions of hydroxide and water, prompting different hydroxide PT pathways and the properly presolvated PT complexes. Solvation configurational probability distributions (Table 3) were calculated as the probability of finding a hydroxide or water molecule that donates *n* HBs and accepts *m* HBs (noted as *n*D*m*A). A geometrical definition of O_*a*_O_*d*_ < 3.4 Å, O_*a*_H_*d*_ < 2.5 Å, and O_*a*_O_*d*_H_*d*_ < 30° of an HB was employed for both water and hydroxide HBs. The distance cutoffs correspond to the first minima of the O–O and O–H RDFs, and the angle cutoff gives a >90% recovery determined by integrating the conditional O_*a*_O_*d*_H_*d*_ angle distribution using these distance cutoffs (see Section S3 of the Supporting Information). Solvation configurational populations at the PT transition state (δ_min_ < 0.1 Å) are also calculated in order to investigate the presolvated structures of the PT complex (see Table 4).

**Table 3:** Solvation Configurational Probability Distribution (%)

O*		2A	3A	4A	5A
GN(16,0)	0D	0.2	31.9	67.2	0.3
	1D		0.0	0.3	0.0
GN(17,0)	0D	0.0	7.2	90.0	2.4
	1D	0.0	0.2	0.1	
GN(19,0)	0D	0.2	22.1	52.1	11.3
	1D		4.7	8.0	1.4

O_W_		0A	1A	2A	3A
GN(16,0)	0D	0.1	0.4	0.1	0.0
	1D	3.5	30.0	17.0	1.0
	2D	11.4	29.9	6.6	0.0
GN(17,0)	0D	0.2	0.2	0.1	0.0
	1D	11.1	17.9	15.4	1.4
	2D	8.7	29.7	14.7	0.5
GN(19,0)	0D	0.4	1.2	0.4	0.0
	1D	3.6	22.8	16.0	0.9
	2D	4.6	26.8	21.7	1.5

**Table 4:** Solvation Configurational Probability Distribution (%) at δ_min_ < 0.1 Å

O*		2A	3A	4A	5A
GN(16,0)	0D	2.1	71.1	26.2	
	1D		0.6		
GN(17,0)	0D	0.1	37.6	57.2	1.0
	1D	0.0	3.7	0.4	
GN(19,0)	0D	2.5	49.9	21.3	0.3
	1D		17.9	7.9	0.2

O_W_^(1*)^		1A	2A	3A	4A
GN(16,0)	1D	5.3	71.3	22.4	
	2D	0.1	0.9		
GN(17,0)	1D	1.2	51.6	37.6	1.1
	2D	0.4	6.9	1.2	
GN(19,0)	1D	4.0	51.4	15.1	0.2
	2D	0.4	21.3	7.5	0.2

In the GN(16,0) system, a hydroxide O* configuration 0D4A, i.e., the hyper-coordinated hydroxide, has the highest population (67.2%). At the PT transition state, the dominating O* configuration is 0D3A (71.1%), suggesting that PT in GN(16,0) is associated with the breaking of an accepted HB of the hydroxide, in agreement with the dynamic hyper-coordination mechanism in the dilute bulk solution. However, the occasional donating HB of hydroxide in bulk aqueous solution is almost absent in the GN(16,0) system as indicated by the vanishing probabilities of 1D*m*A hydroxide configurations at both the PT resting state and the PT transition state, and the presolvated hydroxide configuration for PT is the trigonal pyramidal 0D3A (71.1%) instead of the full tetrahedral 1D3A structure (0.6%). Correspondingly, the most active first solvation shell water at the PT transition state is dominated by a 1D2A pattern (71.3%), the configuration also involving a dangling hydrogen. This substantial population of 1D2A at the PT transition state is in stark contrast with the low population (17.0%) of 1D2A for all water molecules in the solution phase, which can be attributed to the asymmetry between water donor and acceptor sites.^96^ Therefore, in addition to breaking an accepting HB of the hydroxide, presolvating the PT complex should also include an extra step in which the proton-donating water adopts the 1D2A configuration. This associated energy cost of this extra step reduces the PT rate and provides an explanation for the suppressed hydroxide diffusivity within GN confinement. Similar suppression of hydroxide transport is observed in a previous study of hydroxide migration in a confined water monolayer between graphene sheets.^97^ In that study, the proton-donating water must also adopt the low population 1D2a pattern to be presolvated for PT to hydroxide.

**Figure 5:**
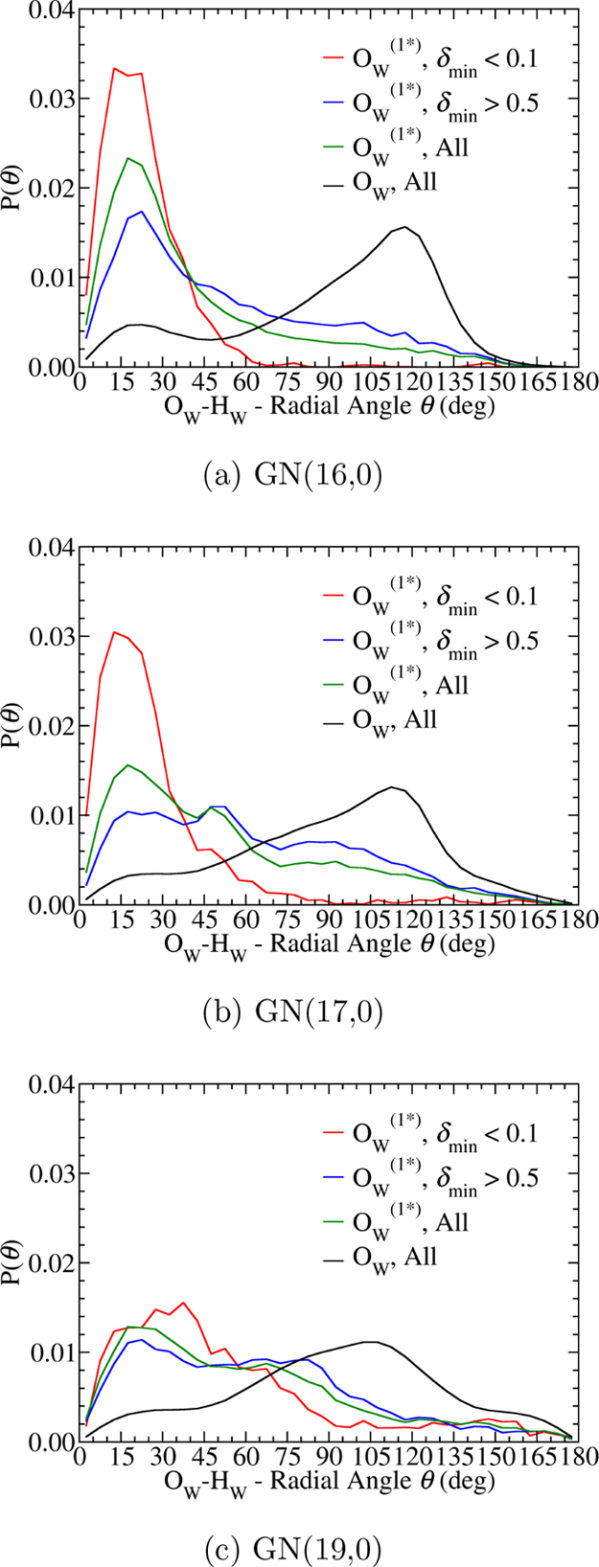
Probability density distributions of the angle θ between O_W_^(1*)^–H_W_ bond of the most active water and the radial vector in (a) GN(16,0), (b) GN(17,0), and (c) GN(19,0). Only the O_W_^(1*)^–H_W_ bond of the most active water not forming a donating hydrogen bond to hydroxide is chosen for calculating the probability density distributions.

As a water molecule assumes the 1D2A solvation configuration, we also observe reorientation of its O–H bond to be aligned along the radial direction and to point to the GN wall. This reorientation is captured in the probability distribution of the O_W_–H_W_ – radial angle θ at different stages of the PT reaction in Figure 5. For the most active first solvation shell water, this angle only pertains to the O_W_^(1*)^–H_W_ bond, which does not donate an HB or participate in PT to the hydroxide. In GN(16,0), this distribution of the most active water at the PT transition state peaks around 20° and the overall profile resembles the distribution of the hydroxide O*–H* – radial angle in Figure 3a. The tail of the distribution, for which θ > 60°, decays rapidly to zero at the PT transition state, clearly indicating the reorientation behavior of this O_W_^(1*)^–H_W_ bond during PT.

A similar dangling hydroxide H* and the reorientation of the proton-donating water O_W_^(1*)^–H_W_ bond also appear in the GN(17,0) system. At the PT transition state, the moderate population (37.6%) of a 0D3A hydroxide solvation pattern and the high population (51.6%) of 1D2A for the most active water suggest that the PT pathway within the same presolvated PT complex in GN(16,0) is still active in GN(17,0). Nevertheless, the high solution density in GN(17,0) creates another PT pathway. The very high population (90.0%) of the 0D4A pattern and low (7.2%) population of the 0D3A hydroxide solvation structure indicate that it is highly energetically unfavorable to break an accepting HB from the hyper-coordinated hydroxide solvation complex. Instead, PT may involve a hyper-coordinated 0D4A hydroxide pattern and a similarly solvated “hyper-coordinated” 1D3A water as evidenced by the high (57.2%) 0D4A population for hydroxide and the second-highest (37.6%) 1D3A population for the most active water at the PT transition state. The hyper-coordinated water, whose oxygen atom has also been referred to as an over-coordinated oxygen (OCO),^96^ has one hydrogen participating in the HB donated to the hydroxide while the other hydrogen dangles. Similar most important or most active PT complexes involving this hyper-coordinated water were also reported in the study of aqueous NaOH solution systems.^95^ This “unconventional” solvation structure of the PT complex involving a hyper-coordinated hydroxide and a hyper-coordinated water provides an explanation for the higher PT reaction free energy barrier, lower PT rate, and further reduced hydroxide diffusivity of the GN(17,0) system. A detailed analysis of the hyper-coordinated water is provided in Section S4 of the Supporting Information.

The dominant solvation configurations in GN(19,0) at the PT transition state are the 0D3A hydroxide pattern (49.9%) and the 1D2A complex for the most active water (51.4%), which are the same configurations as in GN(16,0). The weak HB donated by hydroxide is present in GN(19,0) and participates in PT reactions as indicated by the higher 1D*m*A hydroxide populations at the PT transition state in GN(19,0) than in GN(16,0) and GN(17,0). The corresponding 2D*m*A pattern for the most active water (without a dangling hydrogen) also has higher populations at the PT transition state than the other two systems. The orientational preference of the O_W_^(1*)^–H_W_ bond of the most active water to be aligned along the radial direction is also reduced as the peaks at small angles broaden and probabilities at large angles persist in both the hydroxide O*–H* – radial angle distribution functions in Figure 2a and the O_W_^(1*)^–H_W_ – radial angle distribution functions in Figure 5c. Overall, with the higher water content and larger GN radius, hydroxide PT in GN(19,0) exhibits features of the dynamic hyper-coordination mechanism and more closely resembles dilute bulk aqueous solution behavior than is seen in GN(16,0) and GN(17,0).

### Hydroxide Diffusion Characteristics in the GN Confined System

In addition to the fundamental PT mechanism, other factors, including competition with vehicular diffusion and interaction with the cations, also affect the hydroxide transport. Here we provide further analysis of these factors.

To quantify the relative contribution from structural diffusion and vehicular diffusion, MSD is decomposed into discrete and continuous components according to^5,98^
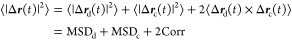
4The bracket represents the ensemble average over all configurations. MSD_d_ and MSD_c_ are the discrete and continuous components corresponding to structural and vehicular diffusion, respectively. The function ⟨Δ***r***_d_(*t*) · Δ***r***_c_(*t*)⟩ is the correlation between these two components. The discrete displacement Δ***r***_d_(*t*) and the continuous displacement Δ***r***_c_(*t*), for the period from time 0 to time *t*, are calculated by accumulating the corresponding elementary displacements δ***r***_d_(*t*, δ*t*) and δ***r***_c_(*t*, δ*t*). If PT occurs during the period from *t* to *t* + δ*t*, the hydroxide displacement during this time window δ*t* is assigned to the elementary discrete displacement δ***r***_d_(*t*, δ*t*) and 0 is assigned to the elementary continuous component δ***r***_c_(*t*, δ*t*). Otherwise, the hydroxide displacement is assigned to the elementary continuous component δ***r***_c_(*t*, δ*t*) and 0 is assigned to the elementary discrete component δ***r***_d_(*t*, δ*t*). The time window δ*t* is selected to be approximately 97 fs, which is close to the 100 fs used in a previous study.^5^ We noticed that the absolute amplitudes of the decomposed MSD curves are affected by the length of the time window, but the relative ordering of the contributions (i.e., discrete > continuous) remains the same (see Section S5 of the Supporting Information).

**Figure 6:**
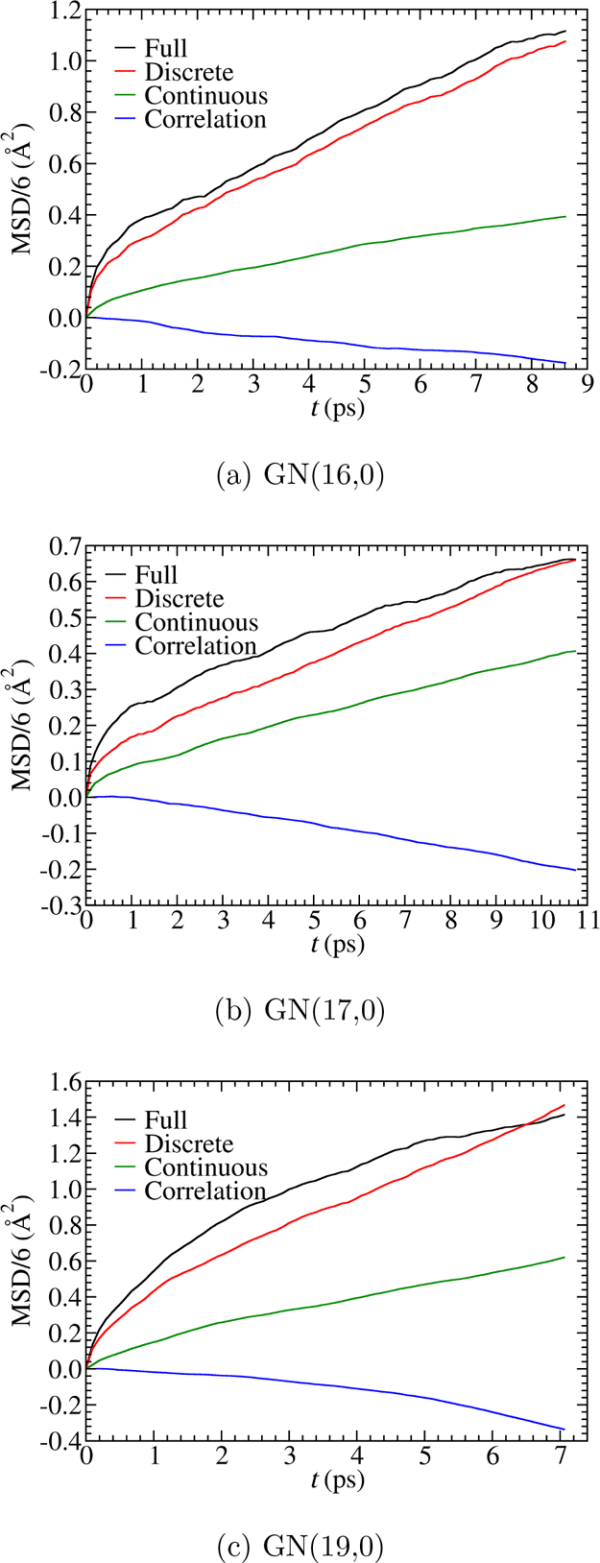
Mean square displacement (MSD) decomposed into discrete and continuous components. The MSD values are divided by 6 so that slopes of the linear regions of the MSD correspond to diffusion coefficients.

As shown in Figure 6, the discrete components of all three systems contribute significantly more than the continuous components, indicating that structural diffusion dominates the hydroxide diffusion mechanism in all three systems. The dominance of structural diffusion in the hydroxide transport process explains why hydroxide diffusivity is maintained at lower water content and lower water diffusivity in GN(16,0). The anticorrelation between discrete and continuous components suggests a competition between structural and vehicular diffusion, which originates from the presence of cations. When the hydroxide hops away from the cation via PT, vehicular diffusion can drag it back toward the cation, and when the hydroxide diffuses away from the cation via the vehicular mechanism, the structural diffusion can bring it back via PT. Similar anticorrelation between the discrete and continuous components was also reported in the MS-EVB simulation of PVBTMA AEM,^5^ while almost zero correlation was observed for an excess proton in aqueous solution confined within a carbon nanotube with no counter ions.^98^

**Figure 7:**
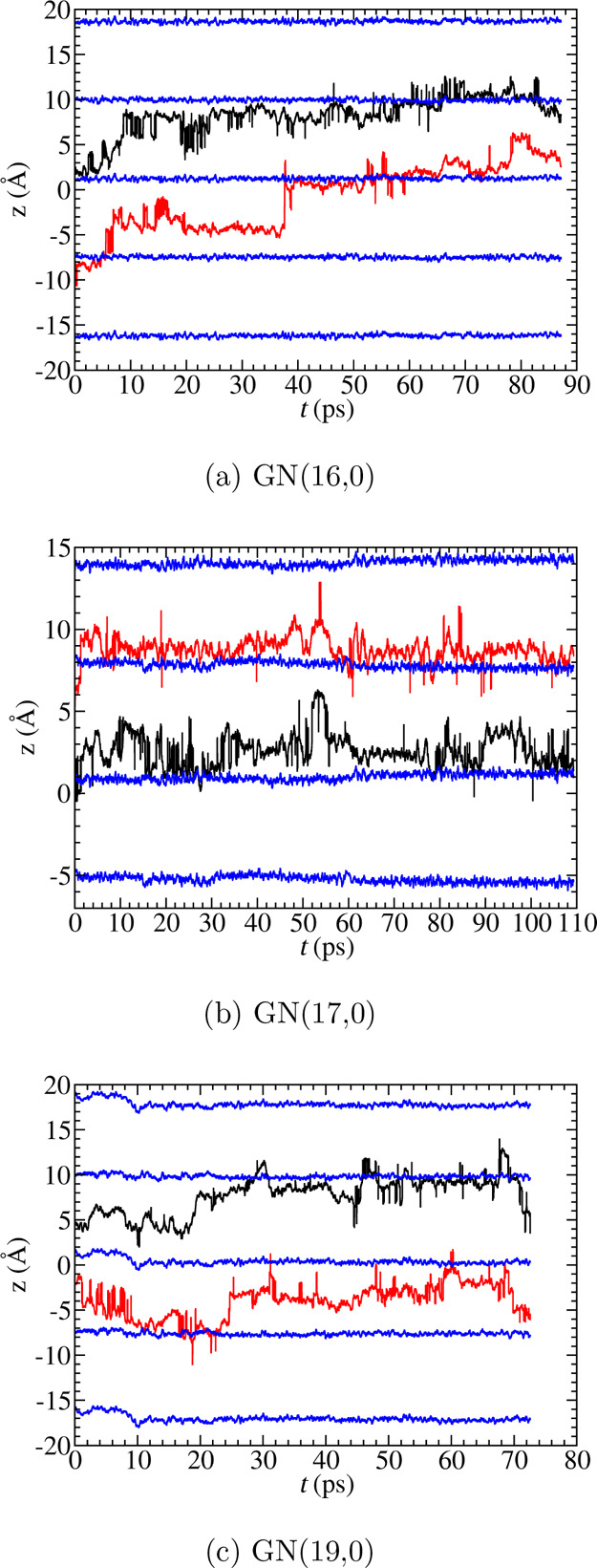
Hydroxide O* (black and red) and cation nitrogen (blue) *z* coordinates at functions of time. The hydroxide coordinates are carefully unwrapped to cross the periodic boundary. Multiple nitrogen replicas are presented.

The strong cation–hydroxide interaction can be illustrated by plotting the *z*-coordinates of the hydroxide O* and cation nitrogen as functions of time (see Figure 7). The figure shows that the hydroxides remain close to one cation within our simulation. One exception is the hydroxide of the red trajectory in GN(16,0) in Figure 7a, which undergoes two successful PT events (∼7 and ∼38 ps) and hops from one cation to the other. The other exception is the hydroxide in the red trajectory of GN(19,0) (Figure 7c), which mainly resides within the region between two cations.

**Figure 8:**
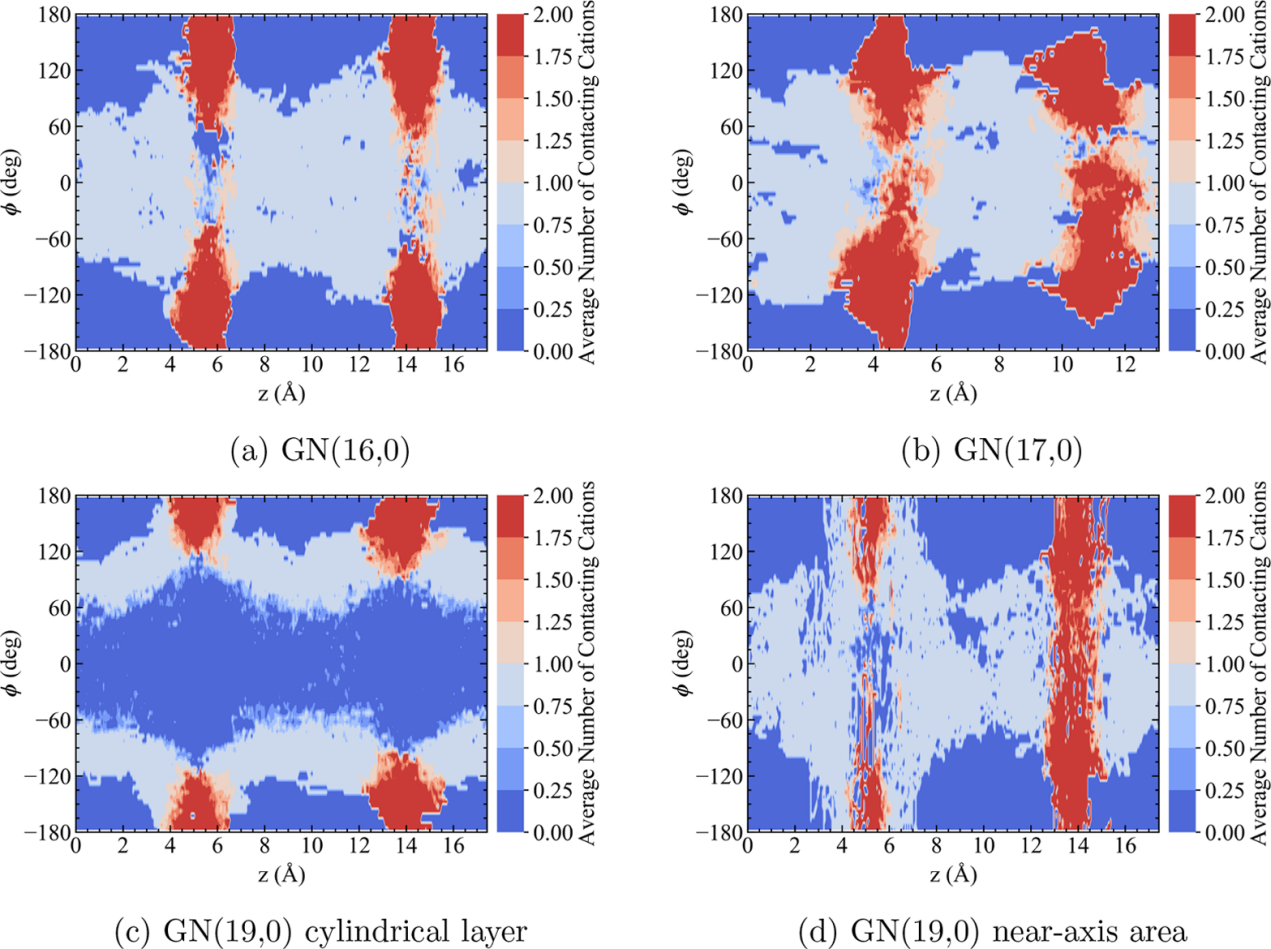
Spatial distributions of the first solvation shells of cations calculated as the average numbers of cation first solvation shells in which an oxygen atom resides (i.e., average numbers of cations that an oxygen atom is contacting with). The angle ϕ is the azimuthal angle with cations attached to ϕ ≈ 0°, and *z* is the axial coordinate. In GN(19,0), a distance cutoff of *r* = 2.175 Å from the minimum of oxygen distributions along radius in Figure 2a is used to distinguish the outer cylindrical layer and the inner near-axis area. Red regions correspond to the overlapping areas between the first solvation shells of two neighboring cations. Gray regions correspond to the nonoverlapping regions of the first solvation shell of only one cation. The blue regions at ϕ = ± 180° are the spaces occupied by cations inside the confinement, while the blue region in (c) at ϕ = 0° corresponds to the solution phase beyond the first solvation shells of any cations.

Further analysis of the hydroxide–cation interaction is performed by dividing the first solvation shells of the TMA cations into two regions—the overlapping region of two first solvation shells of two neighboring cations and the nonoverlapping region of the first solvation shells belonging to just one cation. An N–O distance cutoff of 5.95 Å, corresponding to the first minimum of N–O RDFs (see Figure S7 of the Supporting Information), defines the first solvation shell of TMA cations. The spatial distribution of the overlapping and the nonoverlapping regions of the first solvation shells of cations is illustrated in Figure 8. The blue “voids” centered on ϕ = ± 180° are occupied by the cations. The red strips correspond to the overlapping region, and the white area corresponds to the nonoverlapping region. In GN(16,0) and GN(17,0), the space occupied by the solution under nanoconfinement only consists of overlapping and nonoverlapping regions. In other words, most of the water and hydroxide species are in the first solvation shell of at least one cation in these two systems. The second solvation shells (and beyond) of TMA cations only appear in GN(19,0) as the blue area centered on ϕ = 0° on the outer cylindrical layer (Figure 8c).

In order to make explicit the roles played by the overlapping and nonoverlapping regions of the first solvation shell of TMA cations in hydroxide transport, we calculated population distributions of the hydroxide O* (as well as all oxygen atoms O) in these two regions and listed them in Table 5. In GN(16,0) and GN(17,0), 99% water and hydroxide molecules exist in the first solvation shells of the cations, consistent with the previous analysis of spatial distributions of the overlapping and nonoverlapping regions. The hydroxide defect can visit different oxygen atoms over time under structural diffusion. If we assume that the hydroxide identity obeys a random distribution among all oxygen atoms, the distribution of hydroxide in the overlapping and nonoverlapping regions would be the same as that of all oxygen atoms. Instead, in both systems, the hydroxide O* has a higher population in the nonoverlapping first solvation shell regions than all oxygen atoms. In other words, the overlapping regions appear as barriers to hydroxide transport, consistent with the limited hydroxide transport from the vicinity of one TMA cation to that of the other in GN(16,0) and GN(17,0).

**Table 5:** Hydroxide O* and All Oxygen Populations (%) in the Two Regions of Cation First Solvation Shells

system name	oxygen type	nonoverlapping regions	overlapping regions
GN(16,0)	O	72	27
	O*	96	4
GN(17,0)	O	63	36
	O*	94	5
GN(19,0)	O	47	14
	O*	53	18

The GN(19,0) system has a larger GN radius and a higher water content, and consequently, the confined space within GN(19,0) accommodates an area beyond the first solvation shells of any cations. Compared to GN(16,0) and GN(17,0), the hydroxide O* in GN(19,0) has a decreased population in the nonoverlapping regions and an increased population in the overlapping regions, approaching the distribution of water in these two regions. Therefore, hydroxide diffuses more freely between these two regions in the GN(19,0) system. On the other hand, the total population of hydroxide O* in the first solvation shell of at least one cation is around 71%, while this population of all oxygen atoms is 61%, indicating that the hydroxide dissociation from the cations is still incomplete.

## Conclusions

We constructed nanoconfined aqueous alkaline solution systems within graphene nanotubes functionalized with TMA cations as idealized models of cylindrical pores in AEMs and simulated these systems using AIMD to study how the hydroxide transport is affected by the confined environments. The hydroxide shows decreased diffusivity in all confined systems compared to bulk solution although the structural diffusion dominates the hydroxide transport.

The solution phases inside the GNs adopt the structures of cylindrical layers. Hydroxide ions in the cylindrical layers preferentially align their O–H bonds along the GN radius toward the GN wall instead of donating HBs through their H* sites, indicating the hydrophobic nature of these H* sites. Correspondingly, water molecules in the first solvation shell water of hydroxide donating HBs to the hydroxide O* atoms also possess a dangling hydrogen oriented toward the GN wall during the PT, producing a symmetrically solvated PT complex. This unfavorable solvation configuration of water (donating just one HB) at the PT transition state reduces the PT rate and serves as one factor causing suppression of hydroxide diffusion in these confined systems. While this is the prominent PT pathway in GN(16,0), in GN(19,0), which has a higher water content, PT to the hydroxide ions more closely resembles that in bulk solution. In GN(17,0), the hyper-coordinated hydroxide and the symmetrically solvated hyper-coordinated water also participate in PT under the high solution density, further reducing the PT rate and hydroxide diffusivity in that system. The other factor impeding hydroxide transport is the strong attraction between hydroxide ions and the TMA cations. The hydroxide is preferentially associated with one cation instead of shared between two cations. And the hydroxide does not fully dissociate from the cation even at higher water content in GN(19,0).

Because of the dominance of discrete, structural diffusion over continuous, vehicular diffusion, as indicated in Figure 6, it is natural to ask about the role of nuclear quantum effects. The importance of nuclear quantum effects in bulk hydroxide transport was explored using *ab initio* path integral calculations in ref (36). There, it was seen that while the quantitative picture changes when nuclear quantum effects are included, the basic mechanistic picture does not. While it is difficult to speculate about how these effects would influence the quantitative results obtained here, it is not expected that the comparative picture of the hydroxide transport characteristics would change significantly by their inclusion. Similarly, because of the relative importance of structural diffusion, which is driven largely by local effects such as dynamical solvation patterns and the hydrogen-bond rearrangement, we would not expect the relatively small GN lengths to influence the mechanistic picture presented much as they are not seen to be significant in bulk aqueous solution.^14^

With the understanding of hydroxide transport in the present nanoconfined model systems, we hope to provide insights that are helpful in the design of AEMs with higher hydroxide conductivity. Since the hydroxide diffusivity is suppressed in these confined systems, avoiding the formation of water channel bottlenecks is desired by minimizing the channel size distributions at small radii. If narrow water channels are inevitable, sizes down to our GN(16,0) (∼6.2 Å) are acceptable as the structural diffusion can help retain the hydroxide diffusivity. Changing the cation chemistry might also help enhance the structural diffusion component of the hydroxide transport, which we will investigate in future work. It is also desired to have complete dissociation between hydroxide and cations, which may be achieved by engineering linkers and polymer backbones so that the cations are buried inside the hydrophobic phases and separated from the hydrophilic phases.

In this study, the electronic structure calculations employed in the AIMD simulations involved significant approximations inherent in the BLYP functional. As Table 2 shows, these approximations affect the diffusivity of water at 300 K, causing the ratio of hydroxide to water diffusion constants to be too high. For the system sizes considered here and in our previous work,^23−26,49,51,75^ employing a higher-level functional such as the SCAN meta-GGA functional,^99^ which has been shown to soften the water structure compared to BLYP^100^ and offer some improvements in the details of the solvation patterns of aqueous hydroxide,^101^ would be worth exploring. As an alternative to meta-GGA functionals, hybrid functionals could be employed. However, for the system sizes employed in this study, a time-saving feature, such as the combination of adaptively compressed exchange operator schemes and multiple time-stepping,^102,1025^ would be needed. Ultimately, extending the system size in order to examine more sophisticated and representative models will be desirable, at which point, AIMD simulations will become infeasible, and more efficient approaches such as reactive neural network potentials^103^ become an attractive option that can retain the accuracy of AIMD if they are trained appropriately.

## Supporting Information Available

MSD plots (Section S1); oxygen distributions along the azimuthal angle ϕ and axial coordinate *z* (Section S2); RDFs and angle distributions for defining HB criteria and cation solvation shells (Section S3); detailed analysis of the hyper-coordinated water (Section S4); and decomposition of the MSD into discrete and continuous compositions with a short time window (Section S5) (PDF)

## Notes

AIMD trajectories generated in this study are available on the Tuckerman group GitHub repository: https://github.com/orgs/TuckermanGroup/repositories.

## References

[ref1] Merle G.; Wessling M.; Nijmeijer K. Anion exchange membranes for alkaline fuel cells: A review. J. Membr. Sci. 2011, 377, 1–35. 10.1016/j.memsci.2011.04.043.

[ref2] Pan J.; Chen C.; Zhuang L.; Lu J. Designing Advanced Alkaline Polymer Electrolytes for Fuel Cell Applications. Acc. Chem. Res. 2012, 45, 473–481. 10.1021/ar200201x. 22075175

[ref3] Hickner M. A.; Herring A. M.; Coughlin E. B. Anion exchange membranes: Current status and moving forward. J. Polym. Sci., Part B: Polym. Phys. 2013, 51, 1727–1735. 10.1002/polb.23395.

[ref4] Han K. W.; Ko K. H.; Abu-Hakmeh K.; Bae C.; Sohn Y. J.; Jang S. S. Molecular Dynamics Simulation Study of a Polysulfone-Based Anion Exchange Membrane in Comparison with the Proton Exchange Membrane. J. Phys. Chem. C 2014, 118, 12577–12587. 10.1021/jp412473j.

[ref5] Chen C.; Tse Y.-L. S.; Lindberg G. E.; Knight C.; Voth G. A. Hydroxide Solvation and Transport in Anion Exchange Membranes. J. Am. Chem. Soc. 2016, 138, 991–1000. 10.1021/jacs.5b11951. 26716727

[ref6] Dekel D. R. Review of cell performance in anion exchange membrane fuel cells. J. Power Sources 2018, 375, 158–169. 10.1016/j.jpowsour.2017.07.117.

[ref7] Gottesfeld S.; Dekel D. R.; Page M.; Bae C.; Yan Y.; Zelenay P.; Kim Y. S. Anion exchange membrane fuel cells: Current status and remaining challenges. J. Power Sources 2018, 375, 170–184. 10.1016/j.jpowsour.2017.08.010.

[ref8] Hagesteijn K. F. L.; Jiang S.; Ladewig B. P. A review of the synthesis and characterization of anion exchange membranes. J. Mater. Sci. 2018, 53, 11131–11150. 10.1007/s10853-018-2409-y.

[ref9] Jeon J. Y.; Park S.; Han J.; Maurya S.; Mohanty A. D.; Tian D.; Saikia N.; Hickner M. A.; Ryu C. Y.; Tuckerman M. E.; et al. Synthesis of Aromatic Anion Exchange Membranes by Friedel–Crafts Bromoalkylation and Cross-Linking of Polystyrene Block Copolymers. Macromolecules 2019, 52, 2139–2147. 10.1021/acs.macromol.8b02355.

[ref10] Cohn E. M. NASA’s Fuel Cell Program. In Fuel Cell Systems; Young G. J., Linden H. R., Eds.; 1969; Chapter 1, pp 1–8.

[ref11] de Grotthuss C. J. T. Sur la décomposition de l’eau et des corps qu’elle tient en dissolution à l’aide de l’éctricité galvanique. Ann. Chim. 1806, 1806, 54–74.

[ref12] Atkins P. W. Atkins’ physical chemistry, 8th ed.; Oxford University Press: Oxford, 2006.

[ref13] Marx D. Proton Transfer 200 Years after von Grotthuss: Insights from Ab Initio Simulations. ChemPhysChem 2006, 7, 1848–1870. 10.1002/cphc.200600128. 16929553

[ref14] Marx D.; Chandra A.; Tuckerman M. E. Aqueous Basic Solutions: Hydroxide Solvation, Structural Diffusion, and Comparison to the Hydrated Proton. Chem. Rev. 2010, 110, 2174–2216. 10.1021/cr900233f. 20170203

[ref15] Voth G. A. Computer Simulation of Proton Solvation and Transport in Aqueous and Biomolecular Systems. Acc. Chem. Res. 2006, 39, 143–150. 10.1021/ar0402098. 16489734

[ref16] Agmon N. The Grotthuss mechanism. Chem. Phys. Lett. 1995, 244, 456–462. 10.1016/0009-2614(95)00905-J.

[ref17] Agmon N.; Bakker H. J.; Campen R. K.; Henchman R. H.; Pohl P.; Roke S.; Thämer M.; Hassanali A. Protons and Hydroxide Ions in Aqueous Systems. Chem. Rev. 2016, 116, 7642–7672. 10.1021/acs.chemrev.5b00736. 27314430 PMC7116074

[ref18] Zhang W.; van Duin A. C. T. ReaxFF Reactive Molecular Dynamics Simulation of Functionalized Poly(phenylene oxide) Anion Exchange Membrane. J. Phys. Chem. C 2015, 119, 27727–27736. 10.1021/acs.jpcc.5b07271.

[ref19] Dong D.; Zhang W.; van Duin A. C. T.; Bedrov D. Grotthuss versus Vehicular Transport of Hydroxide in Anion-Exchange Membranes: Insight from Combined Reactive and Nonreactive Molecular Simulations. J. Phys. Chem. Lett. 2018, 9, 825–829. 10.1021/acs.jpclett.8b00004. 29390610

[ref20] Dong D.; Zhang W.; Barnett A.; Lu J.; Van Duin A. C. T.; Molinero V.; Bedrov D. Multiscale Modeling of Structure, Transport and Reactivity in Alkaline Fuel Cell Membranes: Combined Coarse-Grained, Atomistic and Reactive Molecular Dynamics Simulations. Polymer 2018, 10, 1289 10.3390/polym10111289. PMC6401961 30961214

[ref21] Zhang W.; Dong D.; Bedrov D.; van Duin A. C. T. Hydroxide transport and chemical degradation in anion exchange membranes: a combined reactive and non-reactive molecular simulation study. J. Mater. Chem. A 2019, 7, 5442–5452. 10.1039/C8TA10651G.

[ref22] Chen C.; Arntsen C.; Tse Y.-L. S. Simulation study of the effects of phase separation on hydroxide solvation and transport in anion exchange membranes. J. Chem. Phys. 2020, 152, 094903 10.1063/1.5143168. 33480722

[ref23] Zelovich T.; Vogt-Maranto L.; Hickner M. A.; Paddison S. J.; Bae C.; Dekel D. R.; Tuckerman M. E. Hydroxide Ion Diffusion in Anion-Exchange Membranes at Low Hydration: Insights from Ab Initio Molecular Dynamics. Chem. Mater. 2019, 31, 5778–5787. 10.1021/acs.chemmater.9b01824.

[ref24] Zelovich T.; Tuckerman M. E. Water Layering Affects Hydroxide Diffusion in Functionalized Nanoconfined Environments. J. Phys. Chem. Lett. 2020, 11, 5087–5091. 10.1021/acs.jpclett.0c01141. 32515960

[ref25] Zelovich T.; Tuckerman M. E. OH- and H3O+ Diffusion in Model AEMs and PEMs at Low Hydration: Insights from Ab Initio Molecular Dynamics. Membranes 2021, 11, 355 10.3390/membranes11050355. 34066142 PMC8151131

[ref26] Zelovich T.; Vogt-Maranto L.; Simari C.; Nicotera I.; Hickner M. A.; Paddison S. J.; Bae C.; Dekel D. R.; Tuckerman M. E. Non-Monotonic Temperature Dependence of Hydroxide Ion Diffusion in Anion Exchange Membranes. Chem. Mater. 2022, 34, 2133–2145. 10.1021/acs.chemmater.1c03594.

[ref27] Marino M.; Melchior J.; Wohlfarth A.; Kreuer K. Hydroxide, halide and water transport in a model anion exchange membrane. J. Membr. Sci. 2014, 464, 61–71. 10.1016/j.memsci.2014.04.003.

[ref28] Dong D.; Wei X.; Hooper J. B.; Pan H.; Bedrov D. Role of cationic groups on structural and dynamical correlations in hydrated quaternary ammonium-functionalized poly(p-phenylene oxide)-based anion exchange membranes. Phys. Chem. Chem. Phys. 2018, 20, 19350–19362. 10.1039/C8CP02211A. 29993087

[ref29] Zhang N.; Huo J.; Yang B.; Ruan X.; Zhang X.; Bao J.; Qi W.; He G. Understanding of imidazolium group hydration and polymer structure for hydroxide anion conduction in hydrated imidazolium-g-PPO membrane by molecular dynamics simulations. Chem. Eng. Sci. 2018, 192, 1167–1176. 10.1016/j.ces.2018.08.051.

[ref30] Huo J.; Qi W.; Zhu H.; Yang B.; He G.; Bao J.; Zhang X.; Yan X.; Gao L.; Zhang N. Molecular dynamics simulation on the effect of water uptake on hydrogen bond network for OH- conduction in imidazolium-g-PPO membrane. Int. J. Hydrogen Energy 2019, 44, 3760–3770. 10.1016/j.ijhydene.2018.12.090.

[ref31] Dubey V.; Maiti A.; Daschakraborty S. Predicting the solvation structure and vehicular diffusion of hydroxide ion in an anion exchange membrane using nonreactive molecular dynamics simulation. Chem. Phys. Lett. 2020, 755, 137802 10.1016/j.cplett.2020.137802.

[ref32] Luque Di Salvo J.; De Luca G.; Cipollina A.; Micale G. Effect of ion exchange capacity and water uptake on hydroxide transport in PSU-TMA membranes: A DFT and molecular dynamics study. J. Membr. Sci. 2020, 599, 117837 10.1016/j.memsci.2020.117837.

[ref33] Car R.; Parrinello M. Unified Approach for Molecular Dynamics and Density-Functional Theory. Phys. Rev. Lett. 1985, 55, 2471–2474. 10.1103/PhysRevLett.55.2471. 10032153

[ref34] Tuckerman M. E. Ab initiomolecular dynamics: basic concepts, current trends and novel applications. J. Phys.: Condens. Matter 2002, 14, R1297–R1355. 10.1088/0953-8984/14/50/202.

[ref35] Marx D.; Hutter J. Ab Initio Molecular Dynamics: Basic Theory and Advanced Methods; Cambridge University Press, 2010.

[ref36] Tuckerman M. E.; Marx D.; Parrinello M. The nature and transport mechanism of hydrated hydroxide ions in aqueous solution. Nature 2002, 417, 925–929. 10.1038/nature00797. 12087398

[ref37] Zhu Z.; Tuckerman M. E. Ab Initio Molecular Dynamics Investigation of the Concentration Dependence of Charged Defect Transport in Basic Solutions via Calculation of the Infrared Spectrum. J. Phys. Chem. B 2002, 106, 8009–8018. 10.1021/jp020866m.

[ref38] Tuckerman M. E.; Chandra A.; Marx D. Structure and Dynamics of OH-(aq). Acc. Chem. Res. 2006, 39, 151–158. 10.1021/ar040207n. 16489735

[ref39] Chandra A.; Tuckerman M. E.; Marx D. Connecting Solvation Shell Structure to Proton Transport Kinetics in Hydrogen–Bonded Networks via Population Correlation Functions. Phys. Rev. Lett. 2007, 99, 145901 10.1103/PhysRevLett.99.145901. 17930688

[ref40] Tuckerman M. E.; Chandra A.; Marx D. A statistical mechanical theory of proton transport kinetics in hydrogen-bonded networks based on population correlation functions with applications to acids and bases. J. Chem. Phys. 2010, 133, 124108 10.1063/1.3474625. 20886925

[ref41] Iacob C.; Sangoro J. R.; Papadopoulos P.; Schubert T.; Naumov S.; Valiullin R.; Kärger J.; Kremer F. Charge transport and diffusion of ionic liquids in nanoporous silica membranes. Phys. Chem. Chem. Phys. 2010, 12, 13798 10.1039/c004546b. 20824257

[ref42] Habenicht B. F.; Paddison S. J.; Tuckerman M. E. Ab initio molecular dynamics simulations investigating proton transfer in perfluorosulfonic acid functionalized carbon nanotubes. Phys. Chem. Chem. Phys. 2010, 12, 8728 10.1039/c0cp00130a. 20556301

[ref43] Habenicht B. F.; Paddison S. J.; Tuckerman M. E. The effects of the hydrophobic environment on proton mobility in perfluorosulfonic acid systems: an ab initio molecular dynamics study. J. Mater. Chem. 2010, 20, 6342 10.1039/c0jm00253d.

[ref44] Iacob C.; Sangoro J. R.; Kipnusu W. K.; Valiullin R.; Kärger J.; Kremer F. Enhanced charge transport in nano-confined ionic liquids. Soft Matter 2012, 8, 289–293. 10.1039/C1SM06581E.

[ref45] Bankura A.; Chandra A. Hydroxide Ion Can Move Faster Than an Excess Proton through One-Dimensional Water Chains in Hydrophobic Narrow Pores. J. Phys. Chem. B 2012, 116, 9744–9757. 10.1021/jp301466e. 22793519

[ref46] Modestino M. A.; Paul D. K.; Dishari S.; Petrina S. A.; Allen F. I.; Hickner M. A.; Karan K.; Segalman R. A.; Weber A. Z. Self-Assembly and Transport Limitations in Confined Nafion Films. Macromolecules 2013, 46, 867–873. 10.1021/ma301999a.

[ref47] Kusoglu A.; Kushner D.; Paul D. K.; Karan K.; Hickner M. A.; Weber A. Z. Impact of Substrate and Processing on Confinement of Nafion Thin Films. Adv. Funct. Mater. 2014, 24, 4763–4774. 10.1002/adfm.201304311.

[ref48] Clark J. K. II; Habenicht B. F.; Paddison S. J. Ab initio molecular dynamics simulations of aqueous triflic acid confined in carbon nanotubes. Phys. Chem. Chem. Phys. 2014, 16, 16465–16479. 10.1039/C4CP01066C. 24983213

[ref49] Zelovich T.; Long Z.; Hickner M.; Paddison S. J.; Bae C.; Tuckerman M. E. Ab Initio Molecular Dynamics Study of Hydroxide Diffusion Mechanisms in Nanoconfined Structural Mimics of Anion Exchange Membranes. J. Phys. Chem. C 2019, 123, 4638–4653. 10.1021/acs.jpcc.8b10298.

[ref50] Kinsey T.; Glynn K.; Cosby T.; Iacob C.; Sangoro J. Ion Dynamics of Monomeric Ionic Liquids Polymerized In Situ within Silica Nanopores. ACS Appl. Mater. Interfaces 2020, 12, 44325–44334. 10.1021/acsami.0c12381. 32886472

[ref51] Zelovich T.; Winey K. I.; Tuckerman M. E. Hydronium ion diffusion in model proton exchange membranes at low hydration: insights from ab initio molecular dynamics. J. Mater. Chem. A 2021, 9, 2448–2458. 10.1039/D0TA10565A. PMC8151131 34066142

[ref52] Dellago C.; Naor M. M.; Hummer G. Proton Transport through Water-Filled Carbon Nanotubes. Phys. Rev. Lett. 2003, 90, 105902 10.1103/PhysRevLett.90.105902. 12689010

[ref53] Lee S. H.; Rasaiah J. C. Proton transfer and the diffusion of H+ and OH– ions along water wires. J. Chem. Phys. 2013, 139, 124507 10.1063/1.4821764. 24089786

[ref54] Rossi M.; Ceriotti M.; Manolopoulos D. E. Nuclear Quantum Effects in H+ and OH– Diffusion along Confined Water Wires. J. Phys. Chem. Lett. 2016, 7, 3001–3007. 10.1021/acs.jpclett.6b01093. 27440483

[ref55] Lu J.; Barnett A.; Molinero V. Effect of Polymer Architecture on the Nanophase Segregation, Ionic Conductivity, and Electro-Osmotic Drag of Anion Exchange Membranes. J. Phys. Chem. C 2019, 123, 8717–8726. 10.1021/acs.jpcc.9b01165.

[ref56] Barnett A.; Lu J.; Molinero V. Width and Clustering of Ion-Conducting Channels in Fuel Cell Membranes Are Insensitive to the Length of Ion Tethers. J. Phys. Chem. C 2021, 125, 27693–27702. 10.1021/acs.jpcc.1c09097.

[ref57] Barnett A.; Lu J.; Molinero V. Mechanism of Facilitation of Ion Mobility in Low-Water-Content Fuel Cell Membranes. J. Phys. Chem. C 2021, 125, 27703–27713. 10.1021/acs.jpcc.1c09096.

[ref58] Pan J.; Chen C.; Li Y.; Wang L.; Tan L.; Li G.; Tang X.; Xiao L.; Lu J.; Zhuang L. Constructing ionic highway in alkaline polymer electrolytes. Energy Environ. Sci. 2014, 7, 354–360. 10.1039/C3EE43275K.

[ref59] Luo X.; Paddison S. J. DPD simulations of anion exchange membrane: The effect of an alkyl spacer on the hydrated morphology. Solid State Ionics 2019, 339, 115012 10.1016/j.ssi.2019.115012.

[ref60] Zhu Z.; Luo X.; Paddison S. J. DPD simulations of anion exchange membranes functionalized with various cationic groups and associated anions. Solid State Ionics 2019, 340, 115011 10.1016/j.ssi.2019.115011.

[ref61] Luo X.; Liu H.; Bae C.; Tuckerman M. E.; Hickner M. A.; Paddison S. J. Mesoscale Simulations of Quaternary Ammonium-Tethered Triblock Copolymers: Effects of the Degree of Functionalization and Styrene Content. J. Phys. Chem. C 2020, 124, 16315–16323. 10.1021/acs.jpcc.0c03903.

[ref62] Lee M.-T. Designing Anion Exchange Membranes with Enhanced Hydroxide Ion Conductivity by Mesoscale Simulations. J. Phys. Chem. C 2020, 124, 4470–4482. 10.1021/acs.jpcc.9b11566.

[ref63] Spek A. L. Single-crystal structure validation with the program PLATON. J. Appl. Crystallogr. 2003, 36, 7–13. 10.1107/S0021889802022112.

[ref64] Bureekaew S.; Horike S.; Higuchi M.; Mizuno M.; Kawamura T.; Tanaka D.; Yanai N.; Kitagawa S. One-dimensional imidazole aggregate in aluminium porous coordination polymers with high proton conductivity. Nat. Mater. 2009, 8, 831–836. 10.1038/nmat2526. 19734885

[ref65] Jankowska A.; Zalewska A.; Skalska A.; Ostrowski A.; Kowalak S. Proton conductivity of imidazole entrapped in microporous molecular sieves. Chem. Commun. 2017, 53, 2475–2478. 10.1039/C7CC00690J. 28180229

[ref66] Hutter D. M. J.; Alavi A.; Deutsch T.; Bernasconi M.; Goedecker S.; Parrinello M. T. M. CPMD, 2009. https://github.com/CPMD-code,. https://github.com/CPMD-code, (accessed October 05, 2022 from http://www.cpmd.org).

[ref67] Hohenberg P.; Kohn W. Inhomogeneous Electron Gas. Phys. Rev. 1964, 136, B864–B871. 10.1103/PhysRev.136.B864.

[ref68] Kohn W.; Sham L. J. Self-Consistent Equations Including Exchange and Correlation Effects. Phys. Rev. 1965, 140, A1133–A1138. 10.1103/PhysRev.140.A1133.

[ref69] Kohn W. Nobel Lecture: Electronic structure of matter—wave functions and density functionals. Rev. Mod. Phys. 1999, 71, 1253–1266. 10.1103/RevModPhys.71.1253.

[ref70] Becke A. D. Density-functional exchange-energy approximation with correct asymptotic behavior. Phys. Rev. A 1988, 38, 3098–3100. 10.1103/PhysRevA.38.3098. 9900728

[ref71] Lee C.; Yang W.; Parr R. G. Development of the Colle-Salvetti correlation-energy formula into a functional of the electron density. Phys. Rev. B 1988, 37, 785–789. 10.1103/PhysRevB.37.785. 9944570

[ref72] Chen B.; Park J. M.; Ivanov I.; Tabacchi G.; Klein M. L.; Parrinello M. First-Principles Study of Aqueous Hydroxide Solutions. J. Am. Chem. Soc. 2002, 124, 8534–8535. 10.1021/ja020350g. 12121087

[ref73] Chen B.; Ivanov I.; Park J. M.; Parrinello M.; Klein M. L. Solvation Structure and Mobility Mechanism of OHsup–/sup: A Car-Parrinello Molecular Dynamics Investigation of Alkaline Solutions. J. Phys. Chem. B 2002, 106, 12006–12016. 10.1021/jp026504w.

[ref74] Ma Z.; Tuckerman M. E. On the connection between proton transport, structural diffusion, and reorientation of the hydrated hydroxide ion as a function of temperature. Chem. Phys. Lett. 2011, 511, 177–182. 10.1016/j.cplett.2011.05.066.

[ref75] Zelovich T.; Simari C.; Nicotera I.; Dekel D. R.; Tuckerman M. E. The impact of carbonation on hydroxide diffusion in nano-confined anion exchange membranes. J. Mater. Chem. A 2022, 10, 11137–11149. 10.1039/D2TA00830K.

[ref76] Lee H.-S.; Tuckerman M. E. Structure of liquid water at ambient temperature fromiab initio/imolecular dynamics performed in the complete basis set limit. J. Chem. Phys. 2006, 125, 154507 10.1063/1.2354158. 17059272

[ref77] Lee H.-S.; Tuckerman M. E. Dynamical properties of liquid water from ab initio molecular dynamics performed in the complete basis set limit. J. Chem. Phys. 2007, 126, 164501 10.1063/1.2718521. 17477608

[ref78] Ma Z.; Zhang Y.; Tuckerman M. E. iAb initio/i molecular dynamics study of water at constant pressure using converged basis sets and empirical dispersion corrections. J. Chem. Phys. 2012, 137, 044506 10.1063/1.4736712. 22852630

[ref79] von Lilienfeld O. A.; Tavernelli I.; Rothlisberger U.; Sebastiani D. Optimization of Effective Atom Centered Potentials for London Dispersion Forces in Density Functional Theory. Phys. Rev. Lett. 2004, 93, 153004 10.1103/PhysRevLett.93.153004. 15524874

[ref80] Lin I.-C.; Coutinho-Neto M. D.; Felsenheimer C.; von Lilienfeld O. A.; Tavernelli I.; Rothlisberger U. Library of dispersion-corrected atom-centered potentials for generalized gradient approximation functionals: Elements H, C, N, O, He, Ne, Ar, and Kr. Phys. Rev. B 2007, 75, 205131 10.1103/PhysRevB.75.205131.

[ref81] Car R.; Parrinello M. Unified approach for molecular dynamics and density functional theory. Phys. Rev. Lett. 1985, 55, 2471 10.1103/PhysRevLett.55.2471. 10032153

[ref82] Martyna G. J.; Klein M. L.; Tuckerman M. Nosé–Hoover chains: The canonical ensemble via continuous dynamics. J. Chem. Phys. 1992, 97, 2635–2643. 10.1063/1.463940.

[ref83] Halle B.; Karlström G. Prototropic charge migration in water. Part 2.—Interpretation of nuclear magnetic resonance and conductivity data in terms of model mechanisms. J. Chem. Soc., Faraday Trans. 2 1983, 79, 1047–1073. 10.1039/F29837901047.

[ref84] Mills R. Self-diffusion in normal and heavy water in the range 1-45.deg. J. Phys. Chem. 1973, 77, 685–688. 10.1021/j100624a025.

[ref85] Hardy E. H.; Zygar A.; Zeidler M. D.; Holz M.; Sacher F. D. Isotope effect on the translational and rotational motion in liquid water and ammonia. J. Chem. Phys. 2001, 114, 3174–3181. 10.1063/1.1340584.

[ref86] Yeh I.-C.; Hummer G. System-Size Dependence of Diffusion Coefficients and Viscosities from Molecular Dynamics Simulations with Periodic Boundary Conditions. J. Phys. Chem. B 2004, 108, 15873–15879. 10.1021/jp0477147.

[ref87] Alexiadis A.; Kassinos S. Molecular Simulation of Water in Carbon Nanotubes. Chem. Rev. 2008, 108, 5014–5034. 10.1021/cr078140f. 18980342

[ref88] Clark J. K. II; Paddison S. J. Ab initio molecular dynamics simulations of water and an excess proton in water confined in carbon nanotubes. Phys. Chem. Chem. Phys. 2014, 16, 17756–17769. 10.1039/C4CP00415A. 25030323

[ref89] Ma X.; Li C.; Martinson A. B. F.; Voth G. A. Water-Assisted Proton Transport in Confined Nanochannels. J. Phys. Chem. C 2020, 124, 16186–16201. 10.1021/acs.jpcc.0c04493.

[ref90] Muñoz-Santiburcio D.; Marx D. On the complex structural diffusion of proton holes in nanoconfined alkaline solutions within slit pores. Nat. Commun. 2016, 7, 12625 10.1038/ncomms12625. 27550616 PMC4996981

[ref91] Kudin K. N.; Car R. Why Are Water-Hydrophobic Interfaces Charged?. J. Am. Chem. Soc. 2008, 130, 3915–3919. 10.1021/ja077205t. 18311970

[ref92] Cole D. J.; Ang P. K.; Loh K. P. Ion Adsorption at the Graphene/Electrolyte Interface. J. Phys. Chem. Lett. 2011, 2, 1799–1803. 10.1021/jz200765z.

[ref93] Grosjean B.; Bocquet M. L.; Vuilleumier R. Versatile electrification of two-dimensional nanomaterials in water. Nat. Commun. 2019, 10, 1656 10.1038/s41467-019-09708-7. 30971700 PMC6458114

[ref94] Yang S.; Chen M.; Su Y.; Xu J.; Wu X.; Tian C. Stabilization of Hydroxide Ions at the Interface of a Hydrophobic Monolayer on Water via Reduced Proton Transfer. Phys. Rev. Lett. 2020, 125, 156803 10.1103/PhysRevLett.125.156803. 33095625

[ref95] Hellström M.; Behler J. Concentration-Dependent Proton Transfer Mechanisms in Aqueous NaOH Solutions: From Acceptor-Driven to Donor-Driven and Back. J. Phys. Chem. Lett. 2016, 7, 3302–3306. 10.1021/acs.jpclett.6b01448. 27504986

[ref96] Agmon N. Liquid Water: From Symmetry Distortions to Diffusive Motion. Acc. Chem. Res. 2012, 45, 63–73. 10.1021/ar200076s. 21978022

[ref97] Bankura A.; Chandra A. Proton transfer through hydrogen bonds in two-dimensional water layers: A theoretical study based on ab initio and quantum-classical simulations. J. Chem. Phys. 2015, 142, 044701 10.1063/1.4905495. 25637997

[ref98] Selvan M. E.; Keffer D.; Cui S.; Paddison S. Proton transport in water confined in carbon nanotubes: a reactive molecular dynamics study. Mol. Simul. 2010, 36, 568–578. 10.1080/08927021003752887.

[ref99] Sun J.; Ruzsinszky A.; Perdew J. Strongly Constrained and Appropriately Normed Semilocal Density Functional. Phys. Rev. Lett. 2015, 115, 036402 10.1103/PhysRevLett.115.036402. 26230809

[ref100] Zheng L.; Chen M.; Sun Z.; Ko H.-Y.; Santra B.; Dhuvad P.; Wu X. Structural, electronic, and dynamical properties of liquid water by iab initio/i molecular dynamics based on SCAN functional within the canonical ensemble. J. Chem. Phys. 2018, 148, 164505 10.1063/1.5023611. 29716217

[ref101] Liu R.; Zhang C.; Liang X.; Liu J.; Wu X.; Chen M. Structural and dynamic properties of solvated hydroxide and hydronium ions in water from iab initio/i modeling. J. Chem. Phys. 2022, 157, 024503 10.1063/5.0094944. 35840383

[ref102] Mandal S.; Kar R.; Klöffel T.; Meyer B.; Nair N. N. Improving the scaling and performance of multiple time stepping-based molecular dynamics with hybrid density functionals. J. Comput. Chem. 2022, 43, 588–597. 10.1002/jcc.26816. 35147988

[ref1025] Tuckerman M.; Berne B. J.; Martyna G. J. Reversible multiple time scale molecular dynamics. J. Chem. Phys. 1992, 97, 1990 10.1063/1.463137.

[ref103] Unke O. T.; Meuwly M. A reactive, scalable, and transferable model for molecular energies from a neural network approach based on local information. J. Chem. Phys. 2018, 148, 241708 10.1063/1.5017898. 29960298

