# Supplementary material for: Hydroxide Diffusion in Functionalized Cylindrical Nanopores as Idealized Models of Anion Exchange Membrane Environments: An Ab Initio Molecular Dynamics Study
Source: J Phys Chem C Nanomater Interfaces. 2023 Feb 2;127(6):2792–804. doi: 10.1021/acs.jpcc.2c05747 (PMC10034739; doi:10.1021/acs.jpcc.2c05747)
Supplement: Supplementary file 1 — jp2c05747_si_001.pdf [file jp2c05747_si_001.pdf]

Supporting Information: Hydroxide Diffusion in Functionalized Cylindrical Nanopores as Idealized Models of Anion Exchange Membrane Environments: an Ab Initio Molecular Dynamics Study

Zhuoran Long[†] and Mark E. Tuckerman[*,†,‡,¶]

†*Department of Chemistry, New York University, New York, NY 10003, USA*

‡*Courant Institute of Mathematical Science, New York University, New York, NY 10012, USA*

¶*NYU-ECNU Center for Computational Chemistry at NYU Shanghai, 3663 Zhongshan Road North, Shanghai 200062, China*

E-mail: mark.tuckerman@nyu.edu

S1 Mean Square Displacement Plots

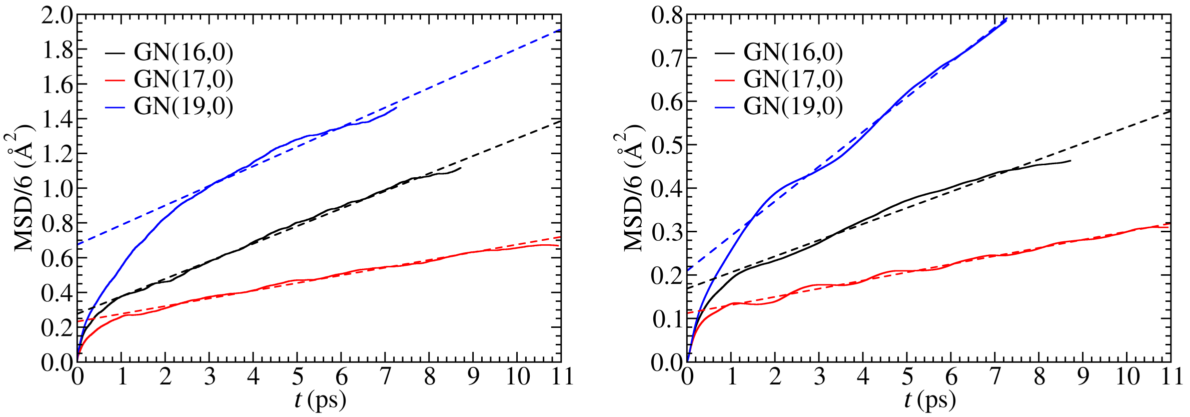

(a) O* MSD (b) O_W MSD

Figure S1: Mean square displacement (MSD) plot of hydroxide O* and water O_W. The MSD curves are divided by 6 so that the slopes of the linear fits equal the diffusion coefficients. The linear fits are performed in the range $t > 1$ ps for GN(16,0) and GN(17,0), and $t > 2$ ps for GN(19,0).

S2 Oxygen Distributions in the Solution Phase

The solution phase oxygen distributions in the cylindrical layers are shown in Figure S2. These distributions are plotted along the azimuthal angle ϕ and axial coordinate z, and averaged over the radial coordinate ρ. Therefore, they can be considered as "flattening" the cylindrical layers. The blue "voids" on $\phi = \pm 180°$ are the space occupied by cations. Oxygen distributions are not uniform in these "incomplete" cylindrical layers of solution phases and instead appear as scattered spots, especially in GN(17,0) with the highest solution-phase density and lowest water diffusion coefficient. The hydroxide O* distributions are plotted in the same way in Figure S3.

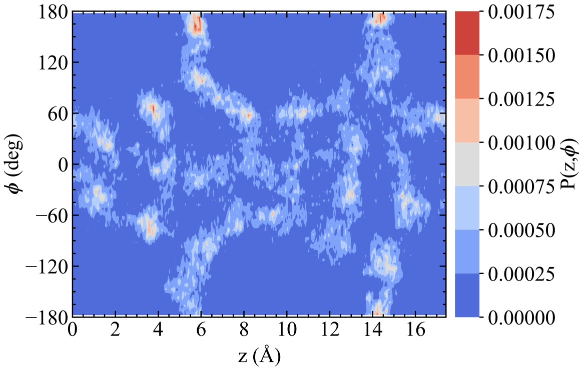

(a) GN(16,0)

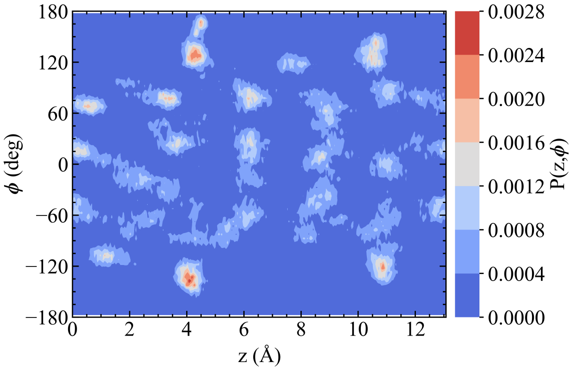

(b) GN(17,0)

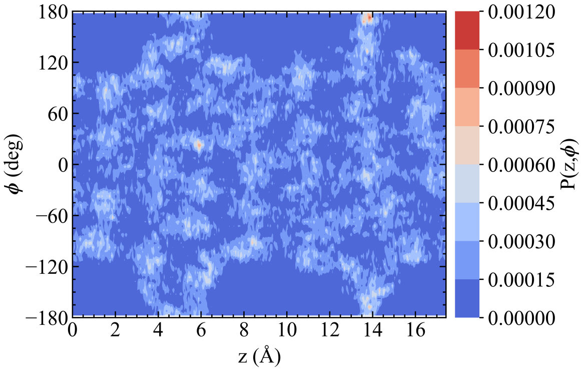

(c) GN(19,0) cylindrical layer

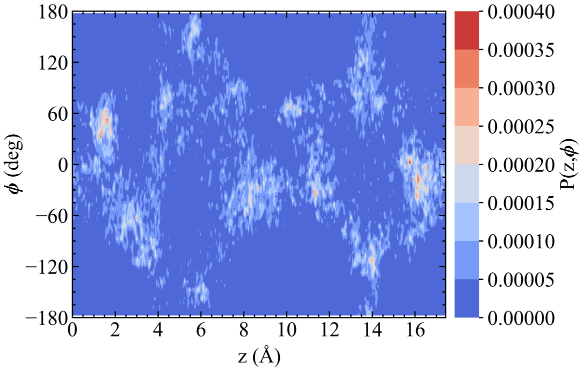

(d) GN(19,0) near-axis area

Figure S2: Spatial distributions of solution phase oxygen atoms along the azimuthal angle ϕ and axial coordinate z (cations are attached to $\phi \approx 0°$). In GN(19,0), a distance cutoff of $r = 2.175$ Å from the minimum of oxygen distributions along radius in Figure 2 of the main text is used to distinguish the outer cylindrical layer and the inner near-axis area.

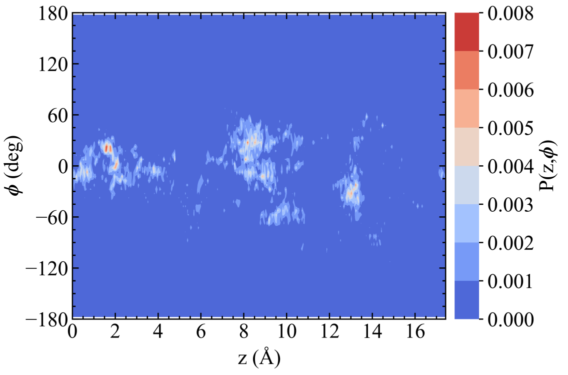

(a) GN(16,0)

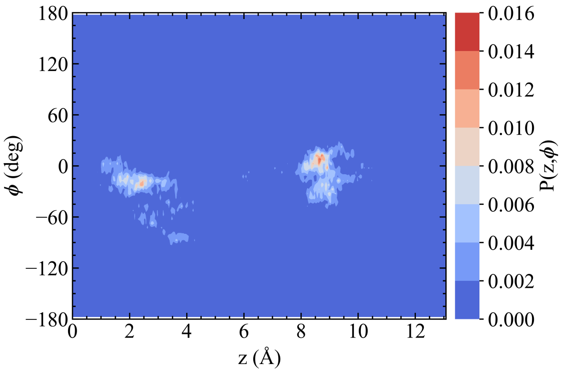

(b) GN(17,0)

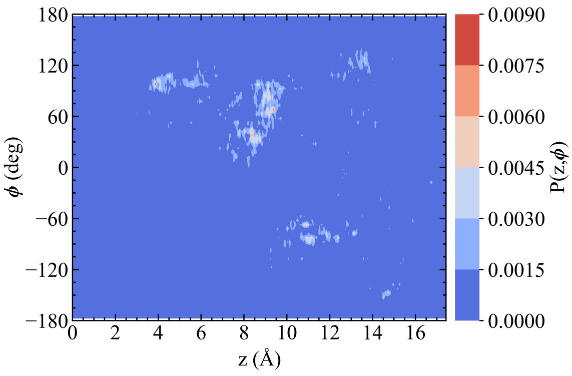

(c) GN(19,0) cylindrical layer

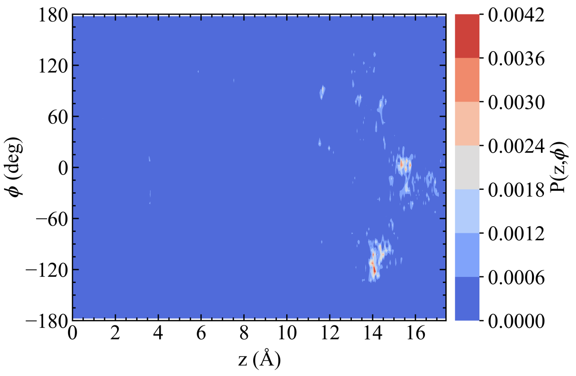

(d) GN(19,0) axis area

Figure S3: Spatial distributions of solution phase hydroxide O* atoms along the azimuthal angle ϕ and axial coordinate z (cations are attached to $\phi \approx 0°$). In GN(19,0), a distance cutoff of $r = 2.175$ Å from the minimum of oxygen distributions along radius in Figure 2 of the main text is used to distinguish the outer cylindrical layer and the inner near-axis area.

S3 Atom Distributions for Geometric Cutoffs

We calculated the radial distribution functions (RDFs) with a bin width of 0.1 Å. For the water-water and water-hydroxide hydrogen bonds (HBs) $O_dH_dO_a$, with O_d being the HB donor oxygen, H_d the HB donor hydrogen, and O_a the HB acceptor oxygen, the O_aO_d and O_aH_d length cutoffs are defined from the first minima of the corresponding RDFs. The $O_aO_dH_d$ angle cutoff gives $> 90\%$ recovery by integrating the conditional distributions of this angle with the length cutoffs to 30°, as listed in Table S1. We also adopt a 5.95 Å cutoff from the N-O RDFs in Figrue S7 to define the first solvation shells of cations.

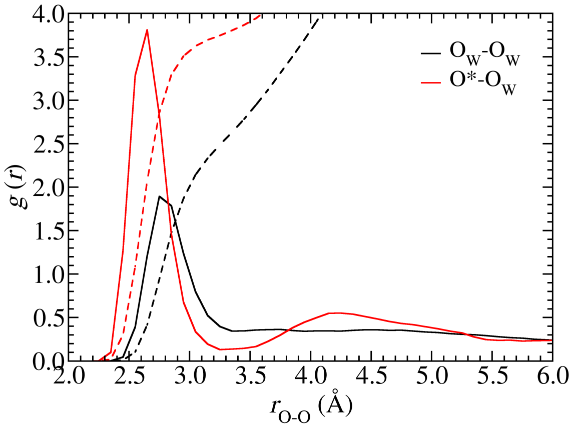

(a) GN(16,0)

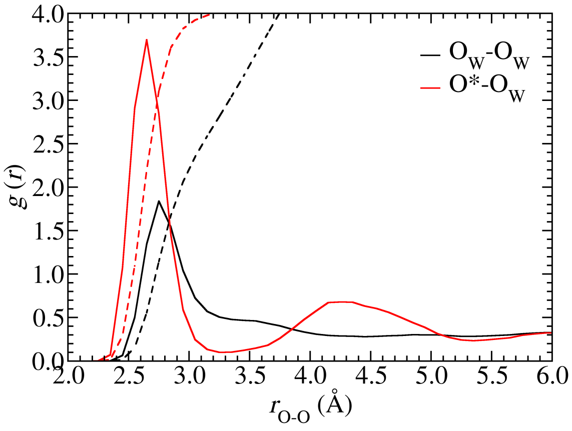

(b) GN(17,0)

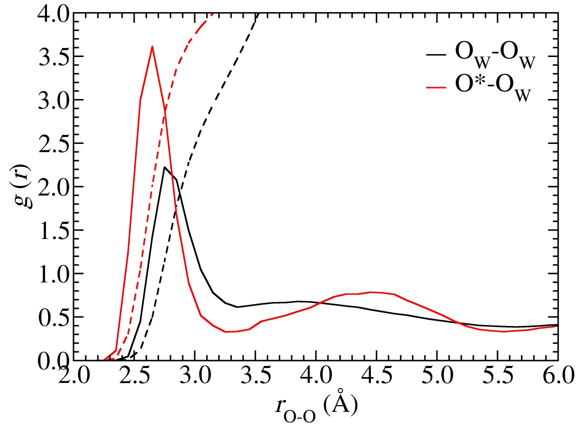

(c) GN(19,0)

Figure S4: Radial distribution functions of water oxygen (O_W) around water and hydroxide (O*)

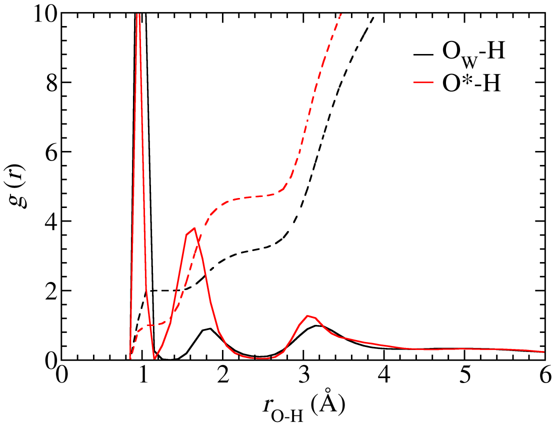

(a) GN(16,0)

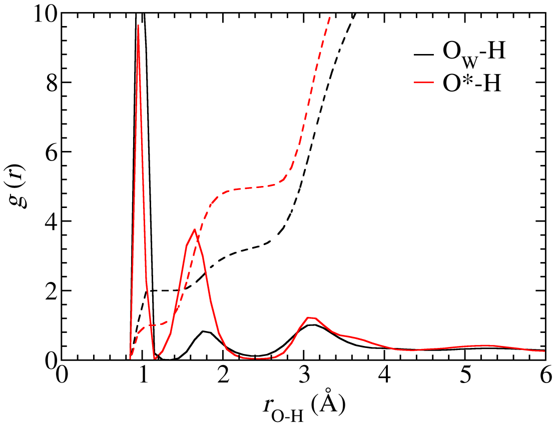

(b) GN(17,0)

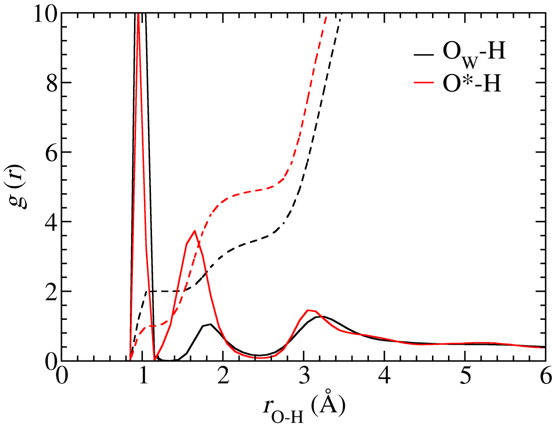

(c) GN(19,0)

Figure S5: Radial distribution functions of solution-phase hydrogen (H) around water (O_W) and hydroxide (O*)

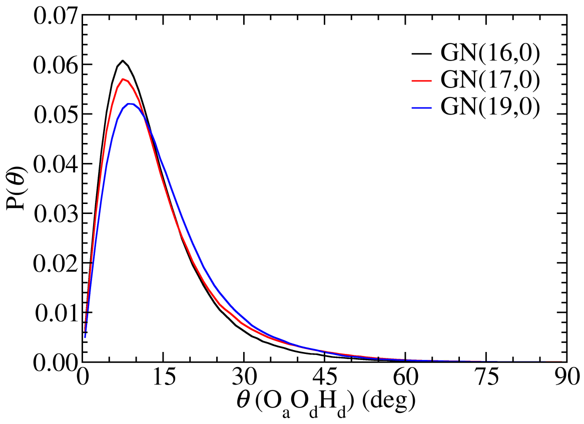

Figure S6: $O_aO_dH_d$ angle distributions with $O_aO_d < 3.4$ Å and $O_aH_d < 2.5$ Å

Table S1: Integral of $O_aO_dH_d$ Angle Distribution to Different Cutoffs

System	Cutoff (°)	Integral	Cutoff (°)	Integral	Cutoff (°)	Integral
GN(16,0)	30	0.943	40	0.981	50	0.994
GN(17,0)	30	0.917	40	0.967	50	0.989
GN(19,0)	30	0.915	40	0.970	50	0.990

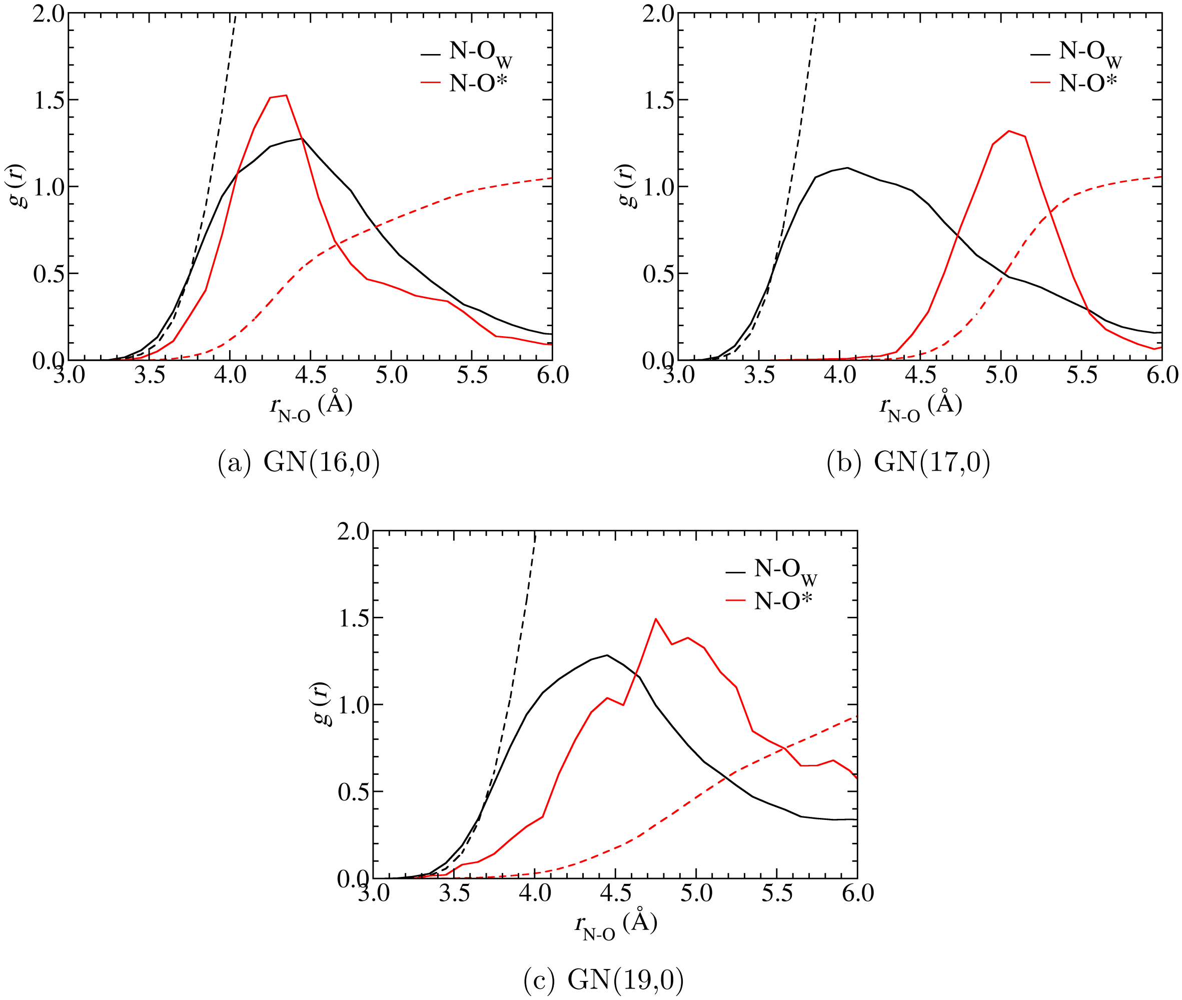

(a) GN(16,0)

(b) GN(17,0)

(c) GN(19,0)

Figure S7: Radial distribution functions of water (O_W) and hydroxide (O^*) around cation nitrogen (N)

S4 Analysis of the Hyper-coordinated Water

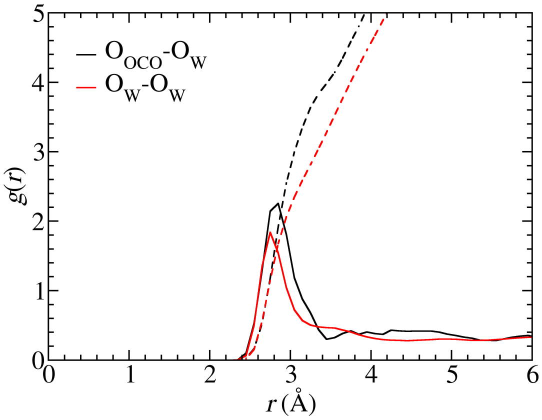

(a) RDF of O_{OCO}-O_W

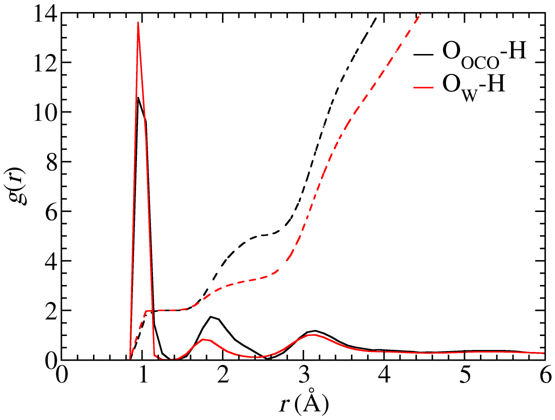

(b) RDF of O_{OCO}-H

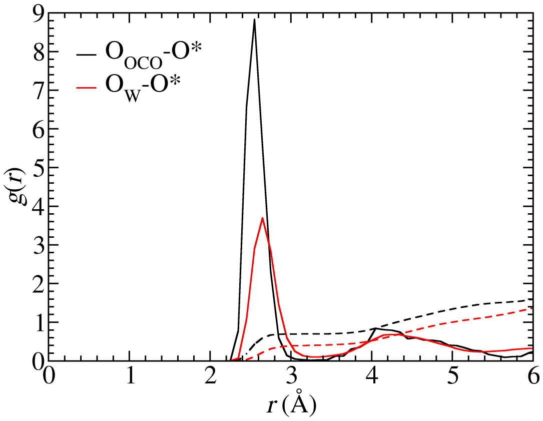

(c) RDF of O_{OCO}-O*

Figure S8: Radial distribution functions centered on the over-coordinated oxygen (OCO) of the hyper-coordinated water. Corresponding water (O_W) radial distribution functions are also plotted for comparison

As discussed in the main text, hydroxide proton transfer (PT) in GN(17,0) is greatly affected by the participation of the hyper-coordinated hydroxide and the symmetrically solvated hyper-coordinated water. To further analyze the structural features of the hyper-coordinated water, we calculate RDFs centering on the over-coordinated oxygen (OCO) and compare them to the RDFs of all water molecules (O_W) in the GM(17,0) system (see Figure S8). The OCOs are defined as water oxygen atoms accepting three HBs, and up to

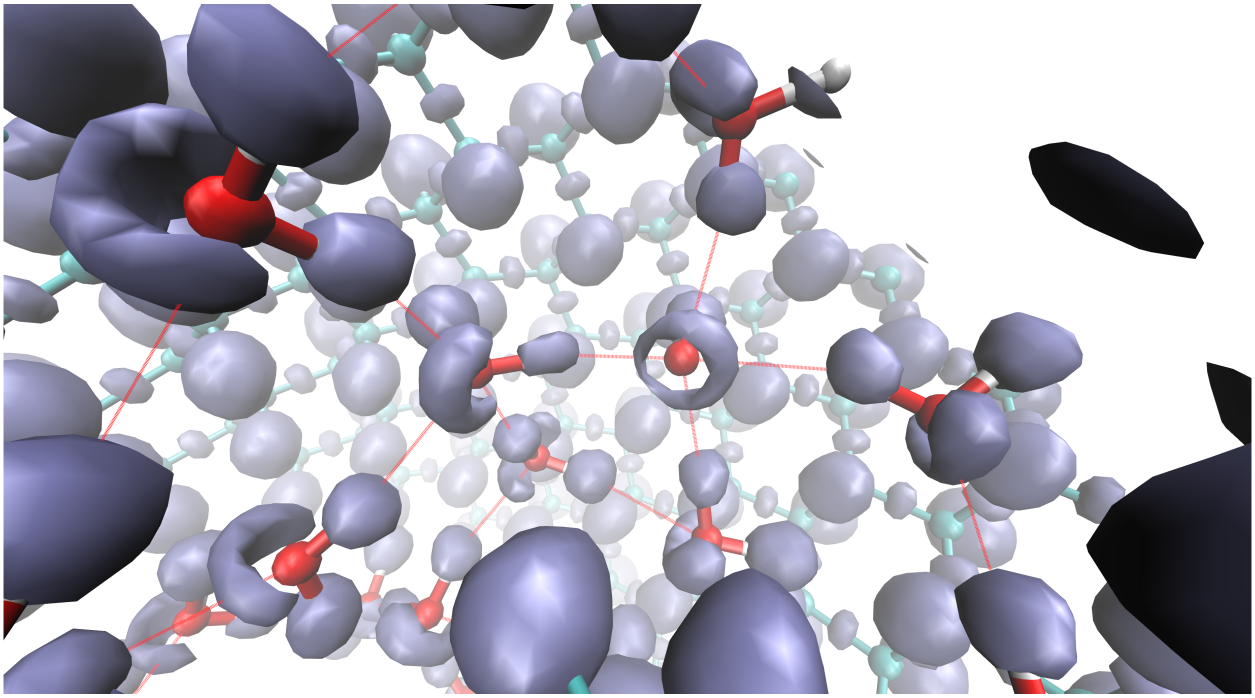

Figure S9: Snapshot of the proton transfer complex involving the hyper-coordinated hydroxide and the hyper-coordinated water, together with the electron localization function (ELF). The ELF cutoff is set to 0.86. Only one periodic replica is presented for visual clarity.

70% water with OCO donates an HB to the hydroxide. The first peak of O_{OCO}-O_W RDF shifts toward a longer distance, indicating a weaker OCO-water HB (HB) than ordinary water-water HBs. The second peak of the O_{OCO}-H RDF, which corresponds to the hydrogen atoms in the OCO's accepting HBs, also shows a similar shift. In contrast, The first peak of O_{OCO}-O* RDF shifts to a closer distance, which can be attributed to the HB shrinking during the PT.

Figure S9 shows an example snapshot of the PT complex involving the hyper-coordinated hydroxide and the hyper-coordinated water, together with the electron localization function (ELF).[1,2] The PT complex is positioned in the middle of the snapshot. The electron lone pairs of the hydroxide have the characteristic ring-shaped ELF at the O* end.[3] The hyper-coordinated water accepts three HBs with an arch-shaped ELF of lone pairs, which is also known as the negativity track.[4] The ring-shaped ELF of the hydroxide is slimmer near the HB with the hyper-coordinated water. The ELF on the transferring hydrogen in that HB

is also smaller than ELFs on other hydrogen atoms in the system. By varying the ELF cutoff isovalue, the arch-shaped ELF of the hyper-coordinated water vanishes at 0.897 while the arch-shaped ELFs on many other water molecules remain, indicating a more dispersed nature of the lone pairs of the hyper-coordinated water.

S5 Decomposition of the Mean Square Displacement

The hydroxide MSD is decomposed into discrete and continuous components following Equation (4) in the main text. The time window δt used in the main text is 4000 a.u. ($\approx$ 97 fs). Here we show MSD curves using the same decomposition method but with a different time window of 40 a.u. ($\approx$ 0.97 fs), which is the output step size of our simulation trajectories. For all three systems, the different size of the time window changes the amplitude of decomposed MSD components but does not change the relative importance of their contributions to the full MSDs nor the anti-correlations between the discrete components and the continuous components.

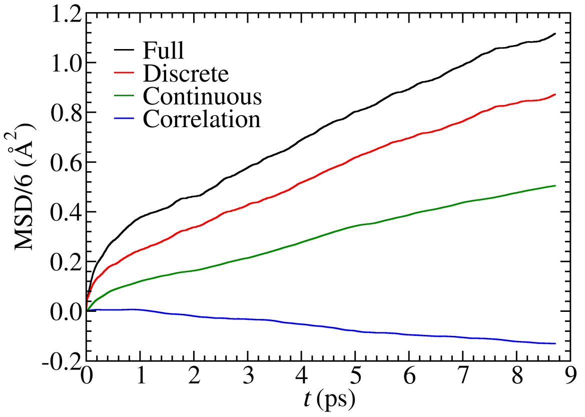

(a) GN(16,0)

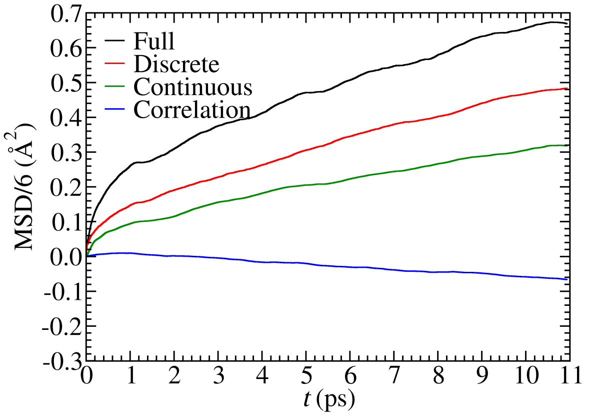

(b) GN(17,0)

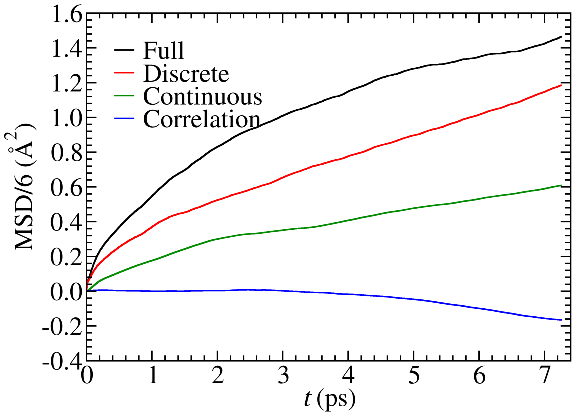

(c) GN(19,0)

Figure S10: Mean square displacement (MSD) decomposed into discrete and continuous components with the time window $\delta t = 40$ a.u. The MSD values are divided by 6 so that the slopes of the linear regions correspond to the diffusion coefficients.

References

(1) Savin, A.; Nesper, R.; Wengert, S.; Fässler, T. F. ELF: The Electron Localization Function. *Angewandte Chemie International Edition in English* **1997**, *36*, 1808–1832.

(2) Becke, A. D.; Edgecombe, K. E. A simple measure of electron localization in atomic and molecular systems. *The Journal of Chemical Physics* **1990**, *92*, 5397–5403.

(3) Marx, D.; Chandra, A.; Tuckerman, M. E. Aqueous Basic Solutions: Hydroxide Solvation, Structural Diffusion, and Comparison to the Hydrated Proton. *Chemical Reviews* **2010**, *110*, 2174–2216.

(4) Agmon, N. Liquid Water: From Symmetry Distortions to Diffusive Motion. *Accounts of Chemical Research* **2012**, *45*, 63–73.
